# TmCactin plays an important role in Gram-negative and -positive bacterial infection by regulating expression of 7 AMP genes in *Tenebrio molitor*

**DOI:** 10.1038/srep46459

**Published:** 2017-04-18

**Authors:** Yong Hun Jo, Yu Jung Kim, Ki Beom Park, Jeong Hwan Seong, Soo Gon Kim, Soyi Park, Mi Young Noh, Yong Seok Lee, Yeon Soo Han

**Affiliations:** 1 Division of Plant Biotechnology, Institute of Environmentally-Friendly Agriculture (IEFA), College of Agriculture and Life Sciences, Chonnam National University, Gwangju 500-757, Republic of Korea; 2 Department of Chemistry and Biochemistry, College of Natural Sciences, California State University, San Bernardino, CA, 92407, USA; 3 Department of Life Science and Biotechnology, College of Natural Sciences, Soonchunhyang University, Asan 336-745, Republic of Korea

## Abstract

Cactin was originally identified as an interactor of the *Drosophila* IκB factor Cactus and shown to play a role in controlling embryonic polarity and regulating the NF-κB signaling pathway. While subsequent studies have identified the roles for Cactin in the mammalian immune response, the immune function of Cactin in insects has not been described yet. Here, we identified a *Cactin* gene from the mealworm beetle, *Tenebrio molitor (TmCactin*) and characterized its functional role in innate immunity. *TmCactin* was highly expressed in prepupa to last instar stages, and its expression was high in the integument and Malpighian tubules of last instar larvae and adults. *TmCactin* was induced in larvae after infection with different pathogens and detectable within 3 hours of infection. The highest levels of *TmCactin* expression were detected at 9 hours post infection. *TmCactin* RNAi significantly decreased the survival rates of larvae after challenge with *Escherichia coli* and *Staphylococcus aureus*, but had no significant effect after challenge with *Candida albicans*. Furthermore, *TmCactin* RNAi significantly reduced the expression of seven antimicrobial peptide genes (AMPs) after bacterial challenge. Our results suggest that TmCactin may serve as an important regulator of innate immunity, mediating AMP responses against both Gram-positive and Gram-negative bacteria in *T. molitor*.

Insects, unlike vertebrates, do not have an adaptive immune response and must rely on their innate immunity for pathogen defense. One important mechanism of innate immunity in insects is the production of antimicrobial peptides (AMPs). These peptides are induced through the activation of two key signaling pathways, namely the Toll and Immune deficiency (Imd) pathways^1^. In *Drosophila* and most other insects, the Toll pathway is activated mainly in response to fungal and Gram-positive bacterial infections^2^. This well-characterized pathway in *Drosophila* results in the phosphorylation and degradation of the inhibitor Cactus, which releases the NF-κB transcription factors Dif and Dorsal into the nucleus for activation of AMP genes such as *Drosomycin*^3^. In contrast, the Imd pathway responds mainly to Gram-negative bacterial infections and promotes nuclear translocation of the NF-κB Relish transcription factor to induce expression of AMP genes such as *Diptericin*^4^,^5^.

In addition to mediating innate immune responses in adult flies, the Toll pathway plays a role in directing *Drosophila* embryonic axis formation^6^. In both processes, the binding of Spätzle activates the Toll receptor, which together with adaptor proteins MyD88, Tube and protein kinase Pelle, causes degradation of Cactus^7^,^8^. Then, depending on the context, the NF-κB proteins Dif and/or Dorsal are released for nuclear translocation^7^,^8^. In the early embryo, degradation of Cactus, which arises in response to developmental cues that activate the Toll pathway, allows Dorsal to translocate into the nucleus and direct the expression of ventral genes, such as *twist, snail*, and *rhomboid*^6^. As mentioned above, the Toll pathway in the *Drosophila* immune response, however, involves the translocation of both Dif and Dorsal to the nucleus where they activate target genes, including AMP genes. It is evident that Dif and Dorsal function redundantly in the regulation of antifungal peptide gene expression in *Drosophila* larvae, but in adults, Dif alone is required for Toll-induced AMP gene transcription^9^,^10^,^11^.

Over the last twenty years, genetic and biochemical analyses in model systems, such as *Drosophila*, nematode, and mammals have led to the characterization of several Toll signaling components. As a result of these studies, it has become more and more apparent that although these components are highly conserved in their sequence and biochemical function, they can have diverse functions. One example of many such components is Cactin, which was initially identified in a yeast two hybrid screen as an interacting protein of *Drosophila* Cactus^12^. Overexpression of Cactin in *Drosophila* embryos causes strong ventralization^12^. Based on these observations and given that Cactus is an inhibitor of Dorsal and that Dorsal functions as a ventral activator, Cactin is suggested to promote nuclear translocation of Dorsal by negatively regulating the stability of the Cactus protein in developing embryos^12^.

Since its discovery in *Drosophila*, Cactin homologs have been identified and functionally characterized in other organisms. In *C. elegans*, Cactin has been shown to be required for normal distal tip cell migration and seam cell proliferation during larval development^13^,^14^. In Arabidopsis, Cactin is necessary for embryogenesis and has been suggested to play a role in splicing based on its interaction with splicesomal proteins in yeast and plant cells^15^. In zebrafish, knockdown of *Cactin* results in developmental defects and embryonic lethality^16^. In *Toxoplasma gondii*, Cactin is suggested to function in cell cycle progression based on the finding that a single mutation in Cactin causes G1 phase arrest^17^. Furthermore, the role of Cactin innate immune responses is suggested by a study showing that in human cells, Cactin overexpression inhibits Toll-like receptor-mediated NF-κB and interferon-regulatory factor (IRF) activation^18^. In further support of this role, the recently cloned *Cactin* from *Litopenaeus vannamei* has been shown to suppress the activities of *Drosophila* and shrimp AMP promoters in *Drosophila* S2 cells^19^.

In the mealworm beetle *Tenebrio molitor*, the upstream mechanisms involved in Toll activation have been elucidated, although relatively little is known about the intracellular signaling events of the *Tenebrio* Toll pathway. So far, it is known that the *Tenebrio* Toll signaling pathway is activated as a result of a three-step proteolytic cascade initiated by pathogen recognition^20^,^21^. Upon binding to lysine-type peptidoglycan from Gram-positive bacteria and diaminopimelic-type peptidoglycan from Gram-negative bacteria, peptidoglycan-recognition protein-SA (PGRP-SA) and Gram-negative binding protein 1 (GNBP1) form a complex that can activate the serine protease cascade and cause cleavage of Spätzle to its active Toll ligand form^20^,^21^. To understand the signaling pathway downstream of the Toll receptor, we recently cloned a myeloid differentiation factor 88 homolog, *TmMyD88*, from *T. molitor* and characterized its gene structure and function in antimicrobial responses^22^. From this study, we found that TmMyD88 contains a typical death domain and a conserved Toll-like interleukin-1 receptor (TIR) domain that can potentially interact with the intracellular TIR domains of TLRs^22^. Furthermore, we found that depletion of *TmMyD88* in *T. molitor* larvae reduced resistance to infection by *S. aureus*, demonstrating that TmMyD88 is required for survival against *Staphylococcus* infection^22^. At the same time as this previous study, Johnston *et al*. conducted an RNA-seq time course analyses of *T. molitor* over a 7-day period in response to bacterial challenge^23^. By comparing their transcriptomic data to the *Tribolium castaneum* predicted proteome, they were able to identify putative orthologs for the majority of the components of the Toll, IMD, and JAK/STAT pathways^23^. For example, among those identified, genes encoding β-1,3-glucan recognition protein and NF-κB transcription factor Relish were transiently induced after immune challenge, while genes encoding AMPs, such as four attacins, and several components of the Toll pathway, including GNBP1, Toll, and serine proteases, SPE and SAE, remained elevated 7 days after challenge^23^.

In this study, we cloned and identified a *Cactin* homolog from *T. molitor*. The temporal and spatial expression profiles of *TmCactin* as well as its response after bacterial and fungal infections were analyzed. Further, we investigated the effects of RNAi-mediated knockdown of *TmCactin* on larval survival and AMP gene expression. Our studies on TmCactin contribute to a better understanding of the Toll/NF-κB pathway regulation and innate immune response in *T. molitor*.

## Results

### Gene organization of *TmCactin* and inferred protein domains

**Figure 1:**
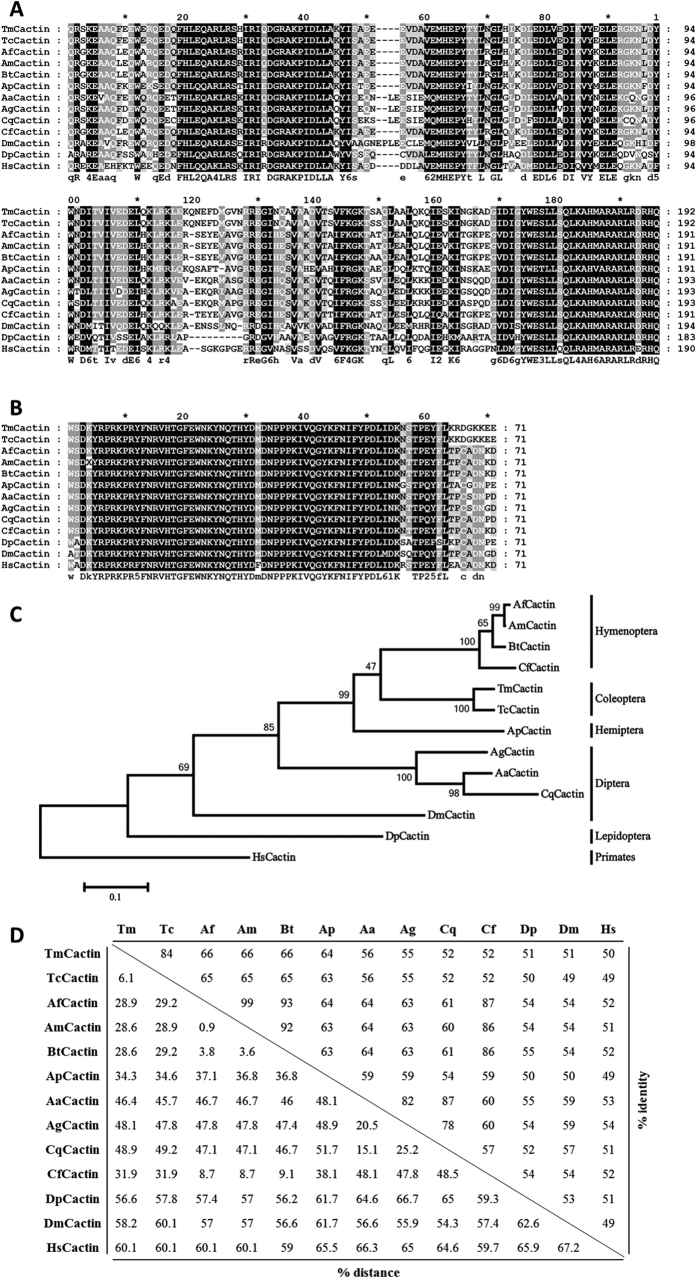
Multiple alignment and phylogenetic analysis of TmCactin. Multiple alignment of the conserved TmCactin mid-region (**A**) and C-terminal Cactus-binding region (**B**). Identical and chemically equivalent amino acid residues are labeled by black or grey underlay, respectively. (**C**) Phylogenetic analysis of TmCactin with representative Cactin from other insects. The analysis was performed using the Maximum Likelihood method based on the JTT matrix-based model and bootstrapped 1,000 times using MEGA6 program. The percentage of trees in which the associated taxa clustered together is shown next to the branches. *Homo sapiens* Cactin sequence were used as an outgroup. (**D**) Percentage identity and distance analysis of TmCactin. TmCactin, *T. molitor* Cactin; TcCactin, *T. castaneum* Cactin (EFA07431.1); DmCactin, *D. melanogaster* Cactin (AAF50904.3); BtCactin, *Bombus terrestris* PREDICTED: uncharacterized protein C19orf29-like isoform 1 (XP_003395486.1); ApCactin; *Acyrthosiphon pisum* Predicted cactin (XP_001952287.2); AfCactin, *Apis florea* Predicted uncharacterized protein C19orf29-like (XP_003696278.1); AmCactin, *Apis mellifera* Predicted cactin isoform 2 (XP_624972.3); AaCactin, *Aedes aegypti* hypothetical protein AaeL_AAEL013167 (XP_001663347.1); CqCactin, *Culex quinquefasciatus* Cactin (XP_001844871.1); CfCactin; *Camponotus floridanus* Uncharacterized protein C19orf29 (EFN62654.1), AgCactin, *Anopheles gambiae* str. PEST AGAP006542-PA (XP_316580.4), DpCactin; *Danaus plexippus* hypothetical protein KGM_02089 (EHJ64422.1); HsCactin; *H. sapiens* Cactin (NP_067054.1).

The *TmCactin* gene was identified by performing local-tblastn searches against the *Tenebrio* RNAseq and EST database using the *T. castanuem* Cactin protein sequence as query. The genomic organization of the *TmCactin* gene was determined by local-blastn against the *Tenebrio* genome database. Comparison between the genomic sequence and cDNA revealed that *TmCactin* (Accession number; KY618833) has seven exons and six introns. The ORF of 2,833 bp encodes a 952 amino acid long polypeptide with a molecular weight of approximately 110 kDa (Figs S1–2). Conserved domain analysis using InterProScan and the NCBI Conserved Domain Database revealed that *TmCactin* has three conserved domains: a coiled-coil domain (also known as the Cactin mid-domain) (Fig. 1A), a C-terminal Cactus-binding domain (Fig.1B), and a C2HC zinc-finger domain conforming to the consensus sequence CX_2–4_CX_3_FX_5_LX_2_HX_3–4_^24^. Furthermore, we performed a blastp search against the NCBI non-redundant protein database using the entire *Tm*Cactin protein sequence and identified numerous homologs in other insects and animals. Of the top 100 hits, we retrieved homologous sequences with amino acid identities in the 56% to 86% range from 57 insect species, including 4 from beetles, 28 from ants, 7 from bees, 6 from wasps, and 2 from *Drosophila* (see Supplementary Table 1). We also retrieved predicted Cactin sequences from five mammals (dolphin, whale, common shrew, prarie vole, and camel), two crustaceans (water flea and shrimp), two alligator species, and three birds. All of these proteins possess a coiled-coil domain and a Cactus-binding domain, but only three of the proteins (uncharacterized proteins KYM93587.1, KYM76580.1, and EFN62654.1) contain multiple zinc-finger domains.

Further phylogenetic analysis was carried out using amino acid sequences from 13 different insect species (with the human Cactin sequence as an outgroup). The phylogenetic tree was generated by MEGA6 using the neighbor-joining algorithm. As expected, insect Cactins from the same insect order were grouped together, with TmCactin and TcCactin on the same branch (Fig. 1C). Furthermore, percent identity and distance data revealed that TmCactin had the highest identity with TcCactin (84% identity) and more than 50% identity with Cactins from other insect orders (66% with Hymenoptera, 64% with Hemiptera, 51–56% with Diptera, and 51% with Lepidoptera) (Fig. 1D).

### Temporal and spatial expression patterns of *TmCactin*

**Figure 2:**
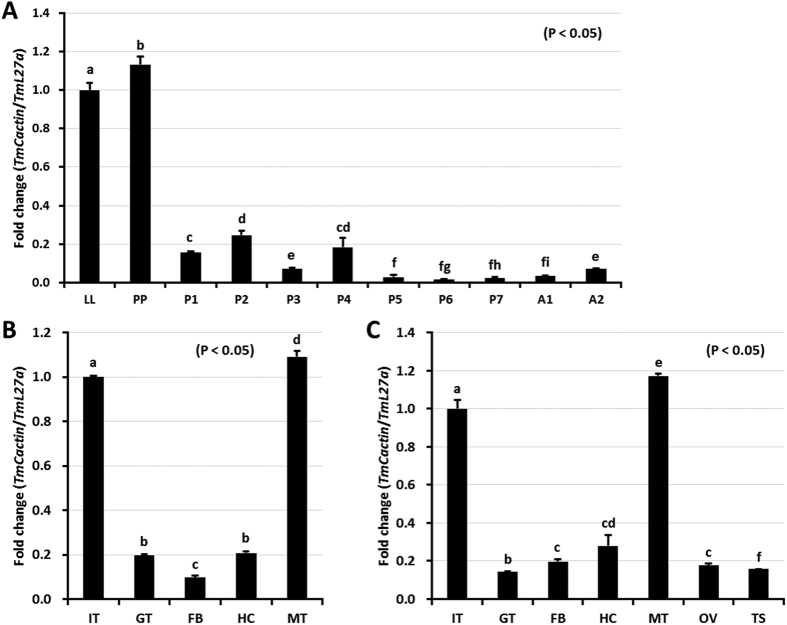
Developmental and tissue specific expression patterns of *TmCactin*. (**A**) The result of developmental expression patterns shows that *TmCactin* is highly expressed in last instar larval and prepupal stages. LL, last instar larva; PP, prepupa; P1-P7, 1–7 day old pupa and A1-A2, 1 and 2 day old adult (**B**) Tissue specific expression patterns indicate that *TmCactin* is highly expressed in integument and Malpighian tubules in both last instar larval and adult stages. Hc, hemocytes; MT, Malpighian tubule; GT, gut; FB, fat body; IT, integument; Ov, ovary and Te, testis. *TmL27a* was used as an internal control and the result was normalized in the last instar larval stage.

To understand the role of TmCactin, we measured its mRNA expression levels at different developmental stages and tissues by qRT-PCR. *TmCactin* was expressed at high levels in last instar larvae and prepupae, but at low levels during the pupal and adult stages (Fig. 2A). Tissue-specific expression analysis showed that *TmCactin* was expressed in all tissues examined, with particularly high expression in the integument and Malpighian tubules of both larvae and adults (Fig. 2B and C).

### Induction pattern analysis of *TmCactin*

**Figure 3:**
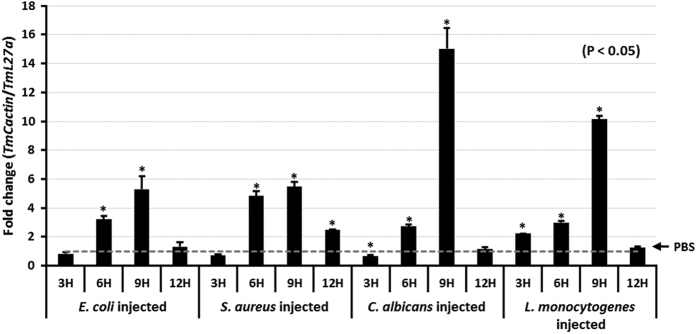
The mRNA expression levels of *TmCactin* after challenge with *E. coli, S. aureus, C. albicans* and *L. monocytogenes*. The relative expression levels were compared with PBS-injected control (shown by a dotted line). The result suggests that *TmCactin* was highly induced 9 h post-infection. *TmL27a* was used as an internal control.

To determine whether *TmCactin* expression can be induced in larvae after fungal and bacterial infections, larvae were injected with either fungi (*C. albicans*), Gram-negative (*E. coli*), or Gram-positive (*S. aureus, L. monocytogenes*) bacteria, and *TmCactin* expression was measured by qRT-PCR at 3, 6, 9 and 12 hours post-infection (Fig. 3). PBS-injected larvae controls were used as a baseline to compare induction of *TmCactin* expression in larvae challenged with bacteria and fungi. We found that after infection with all four microorganisms, *TmCactin* mRNA levels increased between 3 and 9 h post infection and then decreased by 12 h. At the 9 h time point, *TmCactin* expression induced by *C. albicans* and *L. monocytogenes* was 3-fold and 2-fold, respectively, higher than that induced by *E. coli* and *S. aureus*.

### Effects of *TmCactin* gene silencing on *Tenebrio* larval mortality

**Figure 4:**
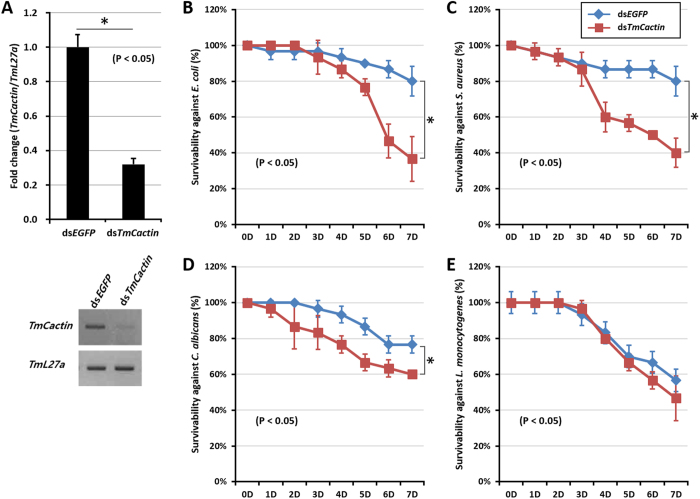
Effect of *TmCactin* gene silencing on survivability of *T. molitor* larvae. (**A**) Down-regulated *TmCactin* transcripts by injection of ds*TmCactin* were measured by qPCR. ds*EGFP*-injected larvae were used as a negative control for RNAi. Survival rates against microbial injection including (**B**) *E. coli*, (**C**) *S. aureus*, and (**D**) *C. albicans* (**E**) *L. monocytogenes* were investigated until 7 days.

Because significant changes in *TmCactin* gene expression were observed following bacterial and fungal infections in *Tenebrio* larvae, we next wanted to test the relevance of TmCactin involvement in immune responses. To assess for this function, we knocked down *TmCactin* expression in 12–15^th^ instar larvae by using dsRNA corresponding to a 599-bp portion of the *TmCactin* gene. After confirming effective knockdown (Fig. 4A), we then examined the survival rates of *TmCactin* RNAi larvae after infection with Gram-negative bacteria (*E. coli*), Gram-positive bacteria (*S. aureus* and *L. monocytogenes*), and fungi (*C. albicans*), respectively, reasoning that if TmCactin were acting as a positive regulator, loss of *TmCactin* would result in increased susceptibility and decreased survival when compared to the control group, while the opposite would be expected if TmCactin was acting as a negative regulator. We observed that knock-down of *TmCactin* leads to reduced larvae survival rates (~50%) compared to the control ds*EGFP* larvae (>80%) six days after infection with *E. coli* (Fig. 4B) or *S. aureus* (Fig. 4C); however, no difference was detected after infection with *C. albicans* (Fig. 4D) and *L. monocytogenes* (Fig. 4E).

### Effects of *TmCactin* gene silencing on *Tenebrio* AMPs expression

**Figure 5:**
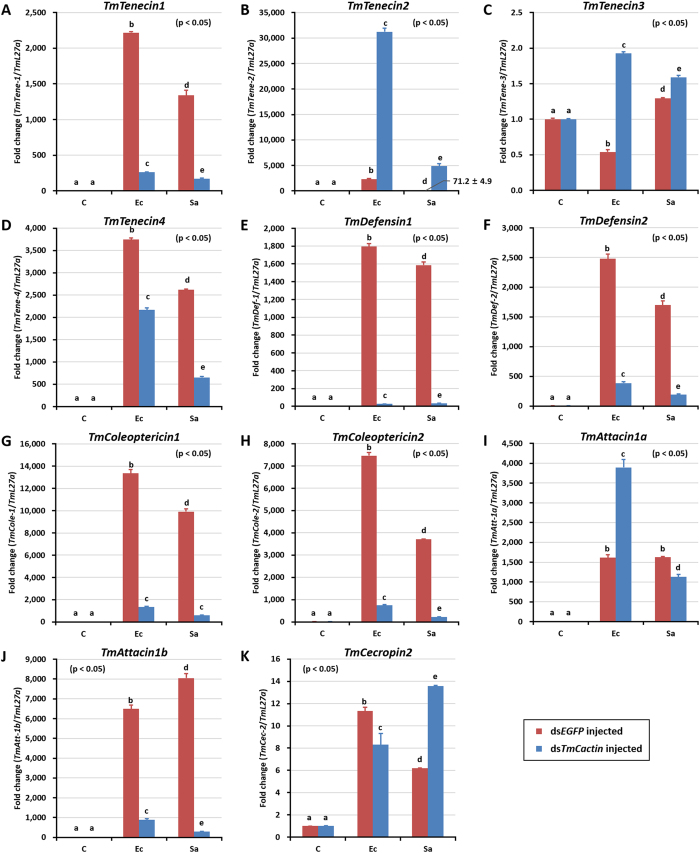
AMP induction patterns against *TmCactin* silenced *T. molitor* larvae in response to pathogenic microbial injection. *E. coli* and *S. aureus* were injected into ds*TmCactin*-teated *T. molitor* larvae and whole body samples were collected at 24 h post injection. Expression of AMP genes including *TmTene-1* (**A**) *TmTenecin-1*), *TmTene-2* (**B**) *TmTenecin-2*), *TmTene-3* (**C**) *TmTenecin-3*), *TmTene-4* (**D**) *TmTenecin-4*), *TmDef-1* (**E**) *TmDefensin-1*), *TmDef-2* (**F**) *TmDefensin-2*), *TmCole-1* (**G**) *TmColeoptericin-1*), *TmCole-2* (**H**) *TmColeoptericin-2*), *TmAtt-1a* (**I**) *TmAttacin-1a*), *TmAtt-1b* (**J**) *TmAttacin-1b*), and *TmCec-2* (**K**) *TmCecropin-2*) were investigated by using qPCR. *EGFP* dsRNA was used as knock down control and *TmL27a* was used as an internal control. (**C**) PBS-injected control; Ec, *E. coli* injected; Sa, *S. aureus* injected.

To investigate the effects of *TmCactin* on AMP gene expression, we measured changes in the mRNA levels of several AMPs in *TmCactin* knockdown larvae following bacterial challenge. As before, we injected larvae with control *EGFP* or *TmCactin* dsRNAs. Then, after 2 days of dsRNA treatment, the larvae were further injected with *E. coli, S. aureus* or PBS, and collected one day later for AMP gene expression analysis (Fig. 5). We found that among the 11 AMPs analyzed, the expression of 9 AMPs was significantly increased (greater than 1,000-fold) in ds*EGFP*-injected larvae after challenge with *E. coli* and *S. aureus* as compared to the PBS control. The fold induction ranged from 1,614-fold (*Att-1a*) to 13,358-fold (*Cole-1*) in response to *E. coli* infection and from 1,585-fold (*Def-1*) to 9,915-fold (*Cole-1*) in response to *S. aureus* infection. Of the 9 AMPs induced by bacterial challenge in ds*EGFP*-injected control larvae, 7 (*Tene-1, Tene-4, Def-1, Def-2, Cole-1, Cole-2*, and *Att-1b*) were significantly reduced in ds*TmCactin*-injected larvae: 45–98% after *E. coli* infection and 75–98% after *S. aureus* infection. Interestingly, in contrast to these observations, we found that *TmCactin* RNAi increased the induction of *Tene-2* (Fig. 5B) and *Atta-1a* (Fig. 5I) by 13.4-fold and 2.4-fold, respectively, after *E. coli* infection. To rule out possible off-target effects, we used a different dsRNA to TmCactin and obtained similar results (Figs S3–S4).

## Discussion

In this study, we identified a Cactin homolog in the coleopteran beetle *T. molitor* and characterized its expression patterns and potential function in innate immunity. *TmCactin* mRNA was found to be highly expressed in late larval and prepupal stages, but at very low levels in the pupal and adult stages. Our tissue expression results indicated that *TmCactin* expression was detectable in all larval tissues examined, but with notable expression in the integument and Malpighian tubules. As in larvae, *TmCactin* expression in adults also appeared to be relatively high in the integument and Malpighian tubules compared with other tissues (gut, fat body, hemocytes, ovaries, and testes). We presume that these differences in *TmCactin* expression between tissues and developmental stages might have to do with hormonal regulation.

We suggest this possibility since several studies in *Drosophila* and other insects have shown that the developmental hormones ecdysone and juvenile hormone regulate the expression of a number of genes involved in innate immune responses^25^,^26^,^27^,^28^,^29^. Interestingly, one of these studies showed that in the *Drosophila* Malpighian tubules of both larvae and adults, AMPs respond to ecdysone very quickly in the absence of infection^28^. In a previous study, these authors showed constitutive expression of AMPs in the Malpighian tubules that persisted from larvae to adults independent of infection^30^. This observation might be explained by the fact that during metamorphosis, Malpighian tubules do not undergo ecdysone-induced destruction as other larval tissues^31^. It is possible that we are seeing a similar hormonal effect on *TmCactin* expression, perhaps resulting from either ecdysone or another developmental hormone, in the Malpighian tubules. Considering the above reasons, and given that components of the IMD^32^ and Toll^33^ pathways, and AMPs^28^,^34^ are expressed in the *Drosophila* Malpighian tubules, it is not surprising that *TmCactin* is highly expressed in these tissues of *T. molitor* larvae and adults. However, it is less clear why *TmCactin* is highly expressed in the larvae and adult integuments. Given that the Toll pathway is required epidermally for muscle development^35^ and that Cactus is necessary for normal neuromuscular function in *Drosophila*^36^, it is possible that TmCactin could be involved in these processes in *T. molitor*. These possibilities need to be examined further.

Furthermore, our expression analysis indicated increased *TmCactin* mRNA expression in the *Tenebrio* larvae after the injection of *E. coli, S. aureus, C. albicans*, and *L. monocytogenes*, with peak levels occurring at 9 hours post-infection. Given this observation, and given that Cactin acts as a positive NF-κB regulator in *Drosophila* development, but as a negative NF-κB regulator of innate immune signaling in shrimp and humans^12^,^18^,^19^, and since there is no existing data on its immune function in insects, we were interested in determining whether TmCactin might be involved in the regulation of innate immune responses in *T. molitor*. We observed that the survival rates of *TmCactin* RNAi larvae were lower compared to the control groups, especially those infected with *E. coli* and *S. aureus*, but this decrease was less significant in those infected with *C. albicans*. These results suggest that TmCactin might have a positive, rather than a negative, role in resistance against Gram-negative and Gram-positive bacterial infections in *T. molitor*. It has been proposed that Cactin in *Drosophila* may play a positive role in the dorsal-ventral pathway in regulating Cactus degradation and Dorsal nuclear translocation^12^. Since the Toll pathway that regulates dorsal-ventral patterning in *Drosophila* embryos also controls the immune response in larvae and adults^37^, we can imagine that Cactin may also positively regulate Dorsal nuclear targeting after immune challenge.

By analogy, we predict that TmCactin could have a similar role in Toll signaling as *Drosophila* Cactin and may regulate the translocation of *Tenebrio* NF-κB-like proteins to the nucleus to activate AMP expression. This hypothesis may be supported by the fact that out of the 213 orthologs identified, several genes related to putative components of the Toll pathway from the beetle, *T. castaneum* were found in *T. molitor*, including Cactus, Dif1, and Dif2^23^. We should also mention that in this same study, an ortholog of Cactin was detected, and although we cannot say for sure, it is possible that the *Cactin* we isolated might be the very same one that Johnston *et al*. (2014) identified in their RNA-seq analyses, since in both cases, they were identified as homologs of *T. castaneum* Cactin, although different search methods were used^23^.

Early studies of *Toll*^*D*^ (gain-of function) mutant and infected wild-type flies revealed that the NF-kB transcription factors Dorsal and Dif are constitutively nuclear and active even in the presence of high levels of Cactus inhibitor^38^,^39^,^40^. To account for this paradox, several explanations have been proposed, one of which is that the nuclear translocation of NF-kB factors is not only correlated to the level of Cactus proteins, but also to the intensity of Cactus degradation^40^. As suggested by Nicolas *et al*, this implies that once dissociated from the Cactus-NF-kB (Dif and/or Dorsal) complex, the NF-kB proteins cannot be inhibited by free Cactus (e.g. because of structural modifications to either or both of the NF-kB and Cactus proteins)^40^. Expanding on this idea, we consider that there may be instances in which Cactus dissociates from the NF-kB complex without being degraded. After dissociating from Dif and Dorsal, a possible scenario might be that Cactus changes its conformation upon binding to Cactin so that it can no longer bind to Dif and Dorsal. We raise this possibility because it has been shown that Cactus expression peaks after 3 hours and levels off thereafter^40^, which suggests to us that Cactus is not fully degraded. Alternatively, Cactus degradation by proteasomes may be promoted by Cactin, which has already been suggested by Lin *et al*. (2000)^12^. Time course experiments could be performed to determine if cactin and cactus gene expression levels are correlated and whether increased levels of Cactin would lead to degradation of Cactus.

Our results, together with previous studies^12^,^18^, indicate that Cactin acts in both *Drosophila* and *T. molitor* as a positive regulator of Toll signaling while in humans, Cactin functions as a negative regulator. While these observations suggest that Cactin is functionally more conserved between *Drosophila* and *Tenebrio* than with human, pairwise sequence alignments of *Drosophila* Cactin (DmCactin) with *Tenebrio* Cactin or human Cactin (hCactin) generates similar identity scores (55% and 52%, respectively), indicating that, sequence-wise, DmCactin is not considerably more similar to TmCactin than it is to hCactin. Yet, despite their overall similarities, close comparison of all three Cactin sequences reveals ten identical residues: N196/127, S198/129, R255/186, N341/268, Q352/279, G404/333, A406/335, S419/348, E512/449, and E571/495 (numbering based on DmCactin and TmCactin, respectively; underlined in Fig. S2) that are shared by DmCactin and TmCactin, but not by hCactin. It is possible that these residues might be of functional importance in distinguishing DmCactin and TmCactin from hCactin.

**Figure 6:**
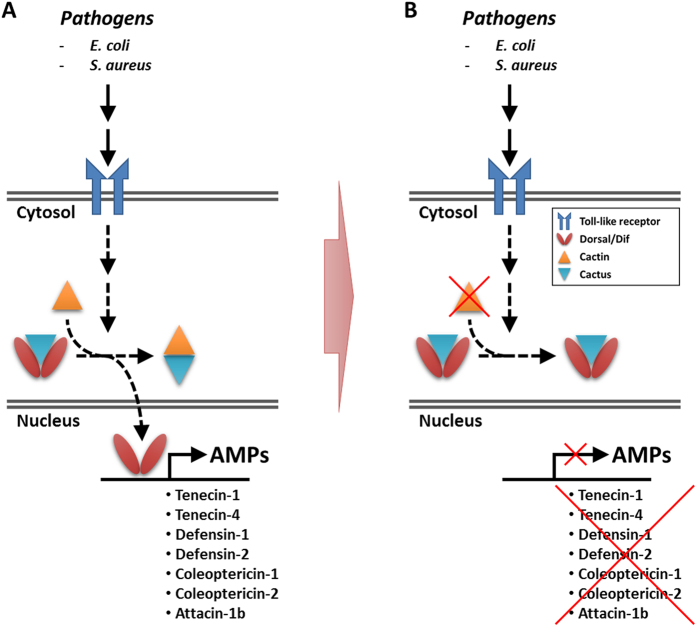
Deduced Toll signaling pathway in *T. molitor*. (**A**) Upon activation of Toll signaling by Gram-positive and Gram-negative bacteria infection, Cactin in the cytosol binds to Cactus and mediates the release of Dif and Dorsal from Cactus, allowing their translocation into the nucleus and activation of AMP genes. (**B**) Depletion of *TmCactin* mRNA causes Dif and Dorsal to be retained in the cytoplasm by Cactus, preventing their entry into the nucleus and activation of AMP gene expression.

Finally, we tested if the expression of fourteen *Tenebrio* AMPs could be induced after *E. coli* and *S. aureus* challenge and whether RNAi knockdown of TmCactin had any effect on the transcription of these AMPs. Bacterial challenge showed that ten of the fourteen AMPs were induced by both *E. coli* and *S. aureus* infections, indicating that these AMPs are involved in the immune responses of *T. molitor* against these two bacteria. Furthermore, of the nine AMPs that responded to bacterial infection, seven of them (Tene1, Tene4, Def1, Def2, Cole1, Cole2, and Atta1b) showed significantly reduced expression after *TmCactin* knockdown. These results, and given that Cactin is an interacting protein of Cactus^12^, and that the *Tenebrio* PGRP-SA can activate the Toll pathway in response to peptidoglycans from both Gram-negative and -positive bacteria^21^, lead us to suggest that in *T. molitor*, TmCactin plays a positive role in the Toll pathway in regulating the expression of these seven AMPs in response to Gram-negative *E. coli* and Gram-positive *S. aureus* (Fig. 6).

Interestingly, however, we also found that *TmCactin* RNAi resulted in increased expression of two AMPs (Tenecin2 and Attacin1a) following *E. coli* challenge, but not *S. aureus* challenge. This suggests the possibility that TmCactin are negative regulators of these two AMPs. In the case of Tenecin2, since it has been demonstrated that production of this AMP is triggered by the Toll pathway through recognition of Gram-negative peptidoglycans^20^,^21^, but shown here to be elevated after *TmCactin* knockdown, it is possible that Tenecin2 may also be activated by other signaling pathways such as the Imd pathway. This leads us to ask if Tenecin2 and other AMPs in *T. molitor* are induced synergistically by both Toll and Imd pathways. Although, as of yet, the Imd pathway in *T. molitor* has not been fully defined, putative orthologs for several components of Imd pathway have been identified in *T. molitor*^23^. It will be interesting to determine in future studies the knockdown effects of these putative *Tenebrio* orthologs on AMP gene expression. This information will help us understand the extent to which the Toll and Imd pathways contribute to the induction of AMP genes in mealworm beetles.

## Materials and Methods

### Insect rearing

The model insect, *Tenebrio molitor*, was maintained at 26 ± 1 °C, 60% ± 5% relative humidity, and under dark conditions with an artificial diet (4.4 g of NeoVita, 0.5 g of chloramphenicol, 0.4 g of L-ascorbic acid, 0.5 g of sorbic acid, 0.5 ml of propionic acid, 2.2 g of yeast extract, 2.2 g of bean powder, 7.6 g of agar, 4.4 g of wheat powder and 73.3 g of wheat bran in 200 ml of D.W; autoclaved at 121 °C for 15 min)^41^. Healthy 12–15^th^ instar larvae were used for all experiments.

### Microorganisms

The *Escherichia coli* K12, *Staphylococcus aureus* RN4220, *Candida albicans* and *Listeria monocytogenes* ATCC7644 were used for this experiment. *E. coli* and *S. aureus* were cultured in Luria-Bertani (LB) broth at 37 °C. *C. albicans* and *L. monocytogenes* were cultured in Sabouraud Dextrose broth and in Brain-heart infusion (BHI) broth at 37 °C, respectively. The microorganisms were harvested and washed in phosphate-buffered saline (PBS; pH 7.0) by centrifugation at 3,500 rpm for 10 min. The washed microorganisms were then suspended in PBS and the concentrations were measured at OD_600_. Finally, 10^6^ cells/μl of *E. coli, S. aureus* and *L. monocytogenes* and 5 × 10^4^ cells/μl of *C. albicans* were used for injection.

### Identification and *in silico* analysis of *TmCactin*

The *TmCactin* was identified by local-tblastn analysis^42^ with *T. castanuem* Cactin amino acid sequences (EFA07431.1) as query and *T. molitor* nucleotide database derived from *Tenebrio* RNAseq/EST (unpublished). To understand the genomic organization of *TmCactin*, local-blastn analysis was performed with obtained nucleotide sequences of *TmCactin* as query and *Tenebrio* genome database (unpublished). Next, the ORF sequences of *TmCactin* were mapped in the genomic sequences. The specific domains were analyzed by using the InterProScan 5^43^,^44^,^45^ and blastp programs. Domain specific multiple alignment was performed with representative Cactin from other insects obtained from Genbank using Clustal X2^46^ and then visualized by using Genedoc software. Phylogenetic analysis and percentage identity and distance analysis were conducted by using the Clustal X2 and MEGA 6 programs^47^. Amino acid sequences of human cactin were used as an outgroup.

### Expression and induction patterns of *TmCactin*

Transcriptomic expression profiles of *TmCactin* were examined by relative quantitative PCR method using the Exicycler Real-Time PCR Quantification System (Bioneer co., Daejon, South Korea). Total RNA was isolated from different developmental stages (last instar larva, prepupa, 1–7 day old pupa, and 1–2 day old adult) and tissues (integument, gut, fat body, Malpighian tubules and hemocytes of last instar larvae and 5-day old adult; ovary and testis of 5-day old adult).

To obtain induction patterns of *TmCactin*, 10^6^ cells/μl of *E. coli, S. aureus* and *L. monocytogenes* and 5 × 10^4^ cells/μl of *C. albicans* were injected into 12–15^th^ instar larvae, respectively, and PBS was used as an injection control. Samples were collected at 3, 6, 9 and 12 h. Total RNA from each sample was isolated by using FavorPrep™ Tri-RNA Reagent (Favorgen biotech corp., Ping-Tung, Taiwan), and 2 μg of total RNAs were used for cDNA synthesis using AccuPower^®^ RT PreMix (Bioneer) with Oligo (dT) 12–18 primer on a MyGenie 96 thermal block (Bioneer). Relative quantitative PCR was performed by using AccuPower^®^ 2X GreenStar qPCR Master Mix (Bioneer) with synthesized cDNAs and specific primers, TmCactin_qPCR_Fw and TmCactin_qPCR_Rv (Table 1) at an initial denaturation of 95 °C for 20 sec, followed by 40 cycles at 95 °C for 5 sec, and 60 °C for 20 sec. *T. molitor ribosomal protein L27a (TmL27a*) was used as an internal control and results were analyzed by using ∆∆Ct methods.

### TmCactin RNAi

599 bp of PCR product was amplified by Ex Taq™ Polymerase (Takara, Japan) with specific primers, TmCactin_Temp_Fw and TmCactin_Temp_Rv (Table 1) at 98 °C for 5 min, followed by 30 cycles at 98 °C for 10 sec, 55 °C for 30 sec and 72 °C for 1 min. Subsequently, the 419 bp PCR product containing the T7 promotor sequences was amplified by Ex Taq™ Polymerase (Takara) (Table 1) in the same condition as mentioned above. Double-stranded RNA (dsRNA) for *TmCactin* was synthesized by using AmpliScribe T7-Flash Transcription Kit (Epicentre, Madison, Wisconsin, USA) and was purified by PCI (Phenol: Chloroform: Isopropyl alcohol mixture) purification, ammonium acetate purification and ethanol precipitation. 2 μg of synthesized ds*TmCactin* was injected into 12–15^th^ instar larvae for gene silencing and the ds*EGFP* was used as a control.

### Mortality assay

To measure mortality, diluted microorganisms (10^6^ cells/μl of *E. coli, S. aureus, L. monocytogenes* and 5 × 10^4^ cells/μl of *C. albicans*) were injected into *TmCactin* silenced *T. molitor* larvae. 10 insects were used for each set of mortality assay and the experiments were triplicated.

### AMP expression patterns

Microorganisms were challenged into *TmCactin* silenced 12–15^th^ instar larva and the samples were collected 12 h post injection. PBS was used as injection control. Total RNAs were isolated and cDNAs were synthesized. A relative quantitative PCR was performed as mentioned above with AMP specific primers (Table 1).

### Statistical analysis

All experiments were performed in triplicate and all data are shown as means ± S.E. The one-way analysis of variance (ANOVA) and Tukey’s multiple range tests were used to evaluate the difference between groups (p < 0.05).

## Additional Information

**How to cite this article:** Jo, Y. H. *et al*. TmCactin plays an important role in Gram-negative and -positive bacterial infection by regulating expression of 7 AMP genes in *Tenebrio molitor. Sci. Rep.* **7**, 46459; doi: 10.1038/srep46459 (2017).

**Publisher's note:** Springer Nature remains neutral with regard to jurisdictional claims in published maps and institutional affiliations.

## Supplementary Material

Supplementary Dataset

## Figures and Tables

**Table 1:** Primers used in the present study.

Name	Primer sequences
TmCactin-Full-Fw	5′-ATGCCAAGAAGTGACCATT-3′
TmCactin-Full-Rv	5′-TCATTCGAACGATTTAAATTTGG-3′
TmCactin_Temp_Fw	5′-ATAAGCGCGGAAGAAGAGGT-3′
TmCactin_Temp_Rv	5′-TCGTTGTCGCTGATTTGTTC-3′
TmCactin_T7_Fw	5′-TAATACGACTCACTATAGGGTACGACATCACGGTCATCGTA-3′
TmCactin_T7_Rv	5′-TAATACGACTCACTATAGGGTCGCCACAGGAGTGGTATTTT-3′
TmCactin#2_T7_Fw	5′-TAATACGACTCACTATAGGGTGACCATTCTCGACGACACAG-3′
TmCactin#2_T7_Rv	5′-TAATACGACTCACTATAGGGTAGTCCTTCCTTGGCCAATTT-3′
TmCactin_qPCR_Fw	5′-AAGCGGCGCAATTTGAAGAG-3′
TmCactin_qPCR_Rv	5′-TCCGCGCTTATGTATTTCGC-3′
TmTene-1_Fw	5′-CAGCTGAAGAAATCGAACAAGG-3′
TmTene-1_Rv	5′-CAGACCCTCTTTCCGTTACAGT-3′
TmTene-2_Fw	5′-CAGCAAAACGGAGGATGGTC-3′
TmTene-2_Rv	5′-CGTTGAAATCGTGATCTTGTCC-3′
TmTene-3_Fw	5′-GATTTGCTTGATTCTGGTGGTC-3′
TmTene-3_Rv	5′-CTGATGGCCTCCTAAATGTCC-3′
TmTene-4_Fw	5′-GGACATTGAAGATCCAGGAAAG-3′
TmTene-4_Rv	5′-CGGTGTTCCTTATGTAGAGCTG-3′
TmDef-1_Fw	5′-AAATCGAACAAGGCCAACAC-3′
TmDef-1_Fw	5′-GCAAATGCAGACCCTCTTTC-3′
TmDef-2_Fw	5′-GGGATGCCTCATGAAGATGTAG-3′
TmDef-2_Fw	5′-CCAATGCAAACACATTCGTC-3′
TmCole-1_Fw	5′-GGACAGAATGGTGGATGGTC-3′
TmCole-1_Rv	5′-CTCCAACATTCCAGGTAGGC-3′
TmCole-2_Fw	5′-GGACGGTTCTGATCTTCTTGAT-3′
TmCole-2_Rv	5′-CAGCTGTTTGTTTGTTCTCGTC-3′
TmAtt-1a_Fw	5′-GAAACGAAATGGAAGGTGGA-3′
TmAtt-1a_Rv	5′-TGCTTCGGCAGACAATACAG-3′
TmAtt-1b_Fw	5′-GAGCTGTGAATGCAGGACAA-3′
TmAtt-1b_Rv	5′-CCCTCTGATGAAACCTCCAA-3′
TmCec-2_Fw	5′-TACTAGCAGCGCCAAAACCT-3′
TmCec-2_Rv	5′-CTGGAACATTAGGCGGAGAA-3′
TmL27a_qPCR_Fw	5′-TCATCCTGAAGGCAAAGCTCCAGT-3′
TmL27a_qPCR_Rv	5′-AGGTTGGTTAGGCAGGCACCTTTA-3′

※ Underlined sequences indicate T7 promotor sequences.

## References

[b1] Hetru C. & Hoffmann J. A. NF-kappaB in the immune response of Drosophila. Cold Spring Harb Perspect Biol 1, a000232, doi: 10.1101/cshperspect.a000232 (2009). 20457557 PMC2882123

[b2] Stokes B. A., Yadav S., Shokal U., Smith L. C. & Eleftherianos I. Bacterial and fungal pattern recognition receptors in homologous innate signaling pathways of insects and mammals. Front Microbiol 6, 19, doi: 10.3389/fmicb.2015.00019 (2015). 25674081 PMC4309185

[b3] Silverman N. . A Drosophila IkappaB kinase complex required for Relish cleavage and antibacterial immunity. Genes Dev 14, 2461–2471 (2000). 11018014 10.1101/gad.817800 PMC316979

[b4] Lemaitre B. & Hoffmann J. The host defense of Drosophila melanogaster. Annu Rev Immunol 25, 697–743, doi: 10.1146/annurev.immunol.25.022106.141615 (2007). 17201680

[b5] De Gregorio E., Spellman P. T., Tzou P., Rubin G. M. & Lemaitre B. The Toll and Imd pathways are the major regulators of the immune response in Drosophila. EMBO J 21, 2568–2579, doi: 10.1093/emboj/21.11.2568 (2002). 12032070 PMC126042

[b6] Belvin M. P. & Anderson K. V. A conserved signaling pathway: the Drosophila toll-dorsal pathway. Annu Rev Cell Dev Biol 12, 393–416, doi: 10.1146/annurev.cellbio.12.1.393 (1996). 8970732

[b7] Lindsay S. A. & Wasserman S. A. Conventional and non-conventional Drosophila Toll signaling. Dev Comp Immunol 42, 16–24, doi: 10.1016/j.dci.2013.04.011 (2014). 23632253 PMC3787077

[b8] Valanne S., Wang J. H. & Ramet M. The Drosophila Toll signaling pathway. J Immunol 186, 649–656, doi: 10.4049/jimmunol.1002302 (2011). 21209287

[b9] Manfruelli P., Reichhart J. M., Steward R., Hoffmann J. A. & Lemaitre B. A mosaic analysis in Drosophila fat body cells of the control of antimicrobial peptide genes by the Rel proteins Dorsal and DIF. EMBO J 18, 3380–3391, doi: 10.1093/emboj/18.12.3380 (1999). 10369678 PMC1171418

[b10] Meng X., Khanuja B. S. & Ip Y. T. Toll receptor-mediated Drosophila immune response requires Dif, an NF-kappaB factor. Genes Dev 13, 792–797 (1999). 10197979 10.1101/gad.13.7.792 PMC316597

[b11] Rutschmann S. . Role of Drosophila IKK gamma in a toll-independent antibacterial immune response. Nat Immunol 1, 342–347, doi: 10.1038/79801 (2000). 11017107

[b12] Lin P. H., Huang L. H. & Steward R. Cactin, a conserved protein that interacts with the Drosophila I kappa B protein Cactus and modulates its function. Mech Develop 94, 57–65, doi: 0.1016/S0925-4773(00)00314-2 (2000). 10.1016/s0925-4773(00)00314-2 10842059

[b13] Tannoury H. . CACN-1/Cactin interacts genetically with MIG-2 GTPase signaling to control distal tip cell migration in C. elegans. Developmental biology 341, 176–185, doi: 10.1016/j.ydbio.2010.02.025 (2010). 20188721 PMC2854247

[b14] LaBonty M. . CACN-1/Cactin Plays a Role in Wnt Signaling in C-elegans. Plos One 9, doi: DOI 10.1371/journal.pone.0101945 (2014). PMC4084952 24999833

[b15] Baldwin K. L., Dinh E. M., Hart B. M. & Masson P. H. CACTIN is an essential nuclear protein in Arabidopsis and may be associated with the eukaryotic spliceosome. Febs Lett 587, 873–879, doi: 10.1016/j.febslet.2013.02.041 (2013). 23454656

[b16] Atzei P., Yang F., Collery R., Kennedy B. N. & Moynagh P. N. Characterisation of expression patterns and functional role of Cactin in early zebrafish development. Gene expression patterns : GEP 10, 199–206, doi: 10.1016/j.gep.2010.03.003 (2010). 20348034

[b17] Szatanek T. . Cactin is essential for G1 progression in Toxoplasma gondii. Mol Microbiol 84, 566–577, doi: 10.1111/j.1365-2958.2012.08044.x (2012). 22486860 PMC3331927

[b18] Atzei P., Gargan S., Curran N. & Moynagh P. N. Cactin targets the MHC class III protein IkappaB-like (IkappaBL) and inhibits NF-kappaB and interferon-regulatory factor signaling pathways. The Journal of biological chemistry 285, 36804–36817, doi: 10.1074/jbc.M110.139113 (2010). 20829348 PMC2978609

[b19] Zhang S. . Molecular characterization and functional analysis of Cactin gene from Litopenaeus vannamei. Fish Shellfish Immunol 41, 608–617, doi: 10.1016/j.fsi.2014.10.014 (2014). 25462455

[b20] Roh K. B. . Proteolytic cascade for the activation of the insect toll pathway induced by the fungal cell wall component. The Journal of biological chemistry 284, 19474–19481, doi: 10.1074/jbc.M109.007419 (2009). 19473968 PMC2740573

[b21] Yu Y. . Diversity of innate immune recognition mechanism for bacterial polymeric meso-diaminopimelic acid-type peptidoglycan in insects. The Journal of biological chemistry 285, 32937–32945, doi: 10.1074/jbc.M110.144014 (2010). 20702416 PMC2963372

[b22] Patnaik B. B. . Gene structure, cDNA characterization and RNAi-based functional analysis of a myeloid differentiation factor 88 homolog in Tenebrio molitor larvae exposed to Staphylococcus aureus infection. Developmental and comparative immunology 46, 208–221, doi: 10.1016/j.dci.2014.04.009 (2014). 24755285

[b23] Johnston P. R., Makarova O. & Rolff J. I nducible defenses stay up late: temporal patterns of immune gene expression in Tenebrio molitor. G3 4, 947–955, doi: 10.1534/g3.113.008516 (2014). PMC4065263 24318927

[b24] Zhang W. . Identification and characterization of DPZF, a novel human BTB/POZ zinc finger protein sharing homology to BCL-6. Biochem Biophys Res Commun 282, 1067–1073, doi: 10.1006/bbrc.2001.4689 (2001). 11352661

[b25] Flatt T. . Hormonal regulation of the humoral innate immune response in Drosophila melanogaster. J Exp Biol 211, 2712–2724, doi: 10.1242/jeb.014878 (2008). 18689425 PMC2522372

[b26] Tian L. . Genome-wide regulation of innate immunity by juvenile hormone and 20-hydroxyecdysone in the Bombyx fat body. BMC Genomics 11, 549, doi: 10.1186/1471-2164-11-549 (2010). 20932328 PMC3091698

[b27] Rus F. . Ecdysone triggered PGRP-LC expression controls Drosophila innate immunity. EMBO J 32, 1626–1638, doi: 10.1038/emboj.2013.100 (2013). 23652443 PMC3671248

[b28] Verma P. & Tapadia M. G. Early gene Broad complex plays a key role in regulating the immune response triggered by ecdysone in the Malpighian tubules of Drosophila melanogaster. Mol Immunol 66, 325–339, doi: 10.1016/j.molimm.2015.03.249 (2015). 25931442

[b29] Sun W., Shen Y. H., Zhou L. X. & Zhang Z. Ecdysone Titer Determined by 3DE-3beta-Reductase Enhances the Immune Response in the Silkworm. J Immunol 196, 1646–1654, doi: 10.4049/jimmunol.1500158 (2016). 26773159

[b30] Verma P. & Tapadia M. G. Immune response and anti-microbial peptides expression in Malpighian tubules of Drosophila melanogaster is under developmental regulation. Plos One 7, e40714, doi: 10.1371/journal.pone.0040714 (2012). 22808242 PMC3395640

[b31] Shukla A. & Tapadia M. G. Differential localization and processing of apoptotic proteins in Malpighian tubules of Drosophila during metamorphosis. Eur J Cell Biol 90, 72–80, doi: 10.1016/j.ejcb.2010.08.010 (2011). 21035895

[b32] McGettigan J. . Insect renal tubules constitute a cell-autonomous immune system that protects the organism against bacterial infection. Insect biochemistry and molecular biology 35, 741–754, doi: 10.1016/j.ibmb.2005.02.017 (2005). 15894191

[b33] Chintapalli V. R., Wang J. & Dow J. A. T. Using FlyAtlas to identify better Drosophila melanogaster models of human disease. Nature Genetics 39, 715–720, doi: 10.1038/ng2049 (2007). 17534367

[b34] Davies S. A. & Dow J. A. Modulation of epithelial innate immunity by autocrine production of nitric oxide. Gen Comp Endocrinol 162, 113–121, doi: 10.1016/j.ygcen.2008.09.012 (2009). 18952086

[b35] Halfon M. S. & Keshishian H. The Toll pathway is required in the epidermis for muscle development in the Drosophila embryo. Developmental biology 199, 164–174, doi: 10.1006/dbio.1998.8915 (1998). 9676200

[b36] Beramendi A., Peron S., Megighian A., Reggiani C. & Cantera R. The inhibitor kappaB-ortholog Cactus is necessary for normal neuromuscular function in Drosophila melanogaster. Neuroscience 134, 397–406, doi: 10.1016/j.neuroscience.2005.04.046 (2005). 15975723

[b37] Wu L. P. & Anderson K. V. Regulated nuclear import of Rel proteins in the Drosophila immune response. Nature 392, 93–97, doi: 10.1038/32195 (1998). 9510254

[b38] Lemaitre B. . Functional analysis and regulation of nuclear import of dorsal during the immune response in Drosophila. EMBO J 14, 536–545 (1995). 7859742 10.1002/j.1460-2075.1995.tb07029.x PMC398111

[b39] Lemaitre B., Nicolas E., Michaut L., Reichhart J. M. & Hoffmann J. A. The dorsoventral regulatory gene cassette spatzle/Toll/cactus controls the potent antifungal response in Drosophila adults. Cell 86, 973–983, doi: 10.1016/S0092-8674(00)80172-5 (1996). 8808632

[b40] Nicolas E., Reichhart J. M., Hoffmann J. A. & Lemaitre B. *In vivo* regulation of the IkappaB homologue cactus during the immune response of Drosophila. The Journal of biological chemistry 273, 10463–10469 (1998). 9553105 10.1074/jbc.273.17.10463

[b41] Jo Y. H. . Molecular characterization and expression analysis of target of rapamycin (TmTOR) in coleopteran insectTenebrio molitor. Entomol Res 46, 139–147, doi: 10.1111/1748-5967.12163 (2016).

[b42] Altschul S. F., Gish W., Miller W., Myers E. W. & Lipman D. J. Basic local alignment search tool. Journal of molecular biology 215, 403–410, doi: 10.1016/S0022-2836(05)80360-2 (1990). 2231712

[b43] Quevillon E. . InterProScan: protein domains identifier. Nucleic Acids Research 33, W116–W120, doi: 10.1093/Nar/Gki442 (2005). 15980438 PMC1160203

[b44] Zdobnov E. M. & Apweiler R. InterProScan - an integration platform for the signature-recognition methods in InterPro. Bioinformatics 17, 847–848, doi: 10.1093/bioinformatics/17.9.847 (2001). 11590104

[b45] Jones P. . InterProScan 5: genome-scale protein function classification. Bioinformatics 30, 1236–1240, doi: 10.1093/bioinformatics/btu031 (2014). 24451626 PMC3998142

[b46] Larkin M. A. . Clustal W and Clustal X version 2.0. Bioinformatics 23, 2947–2948, doi: 10.1093/bioinformatics/btm404 (2007). 17846036

[b47] Tamura K., Stecher G., Peterson D., Filipski A. & Kumar S. MEGA6: Molecular Evolutionary Genetics Analysis version 6.0. Molecular biology and evolution 30, 2725–2729, doi: 10.1093/molbev/mst197 (2013). 24132122 PMC3840312

