# Supplementary material for: TmCactin plays an important role in Gram-negative and -positive bacterial infection by regulating expression of 7 AMP genes in Tenebrio molitor
Source: Sci Rep. 2017 Apr 18;7:46459. doi: 10.1038/srep46459 (PMC5394457; doi:10.1038/srep46459)
Supplement: Supplementary Dataset [file srep46459-s1.pdf]

Supplementary data

TmCactin plays an important role in Gram-negative and -positive bacterial infection by regulating expression of 7 AMP genes in *Tenebrio molitor*

Yong Hun JO[1], Yu Jung Kim[2], Ki Beom PARK[1], Jeong Hwan Seong[1], Soo Gon KIM[1], Soyi PARK[1], Mi Young NOH[1], Yong Seok LEE[3] and Yeon Soo HAN[1*]

Figure S1. Genomic organization and sequence of *TmCactin*. (A) *TmCactin* gene structure has been shown containing the promoter, exon (E1-E7), and intron (I1-I6) regions. Translation start (ATG) and stop codons (TGA) are shown. (B) The gene sequence of *TmCactin* is depicted with the same colors as shown in (A). The *TmCactin* ORF sequences were identified by blast analysis and were aligned with the genomic sequence. *TmCactin* gene contains 7 exons interspersed with 6 introns.

Figure S2. Nucleotide and deduced amino acid sequence of *TmCactin*. *TmCactin* contains an ORF size of 2,833 bp that encodes a protein of 952 amino acids. Domain analysis by InterProScan 5 and blastp shows a conserved Cactin mid-region (open box), a C-terminal Cactus-binding region (gray box), and a zinc-finger domain (black box) in the amino acid sequence of TmCactin. Two regions for dsRNA synthesis are marked by blue (ds*TmCactin#2*) and green (ds*TmCactin*) arrows, and red arrows indicate the region for qPCR primers.

Figure S3. Validation experiments using a second *TmCactin* dsRNA. (A) Quantitative RT-PCR shows significant reduction in *TmCactin* mRNA in larvae injected with dsRNA against *TmCactin* (ds*TmCactin* #2) compared to *EGFP* dsRNA control-injected larvae. (B) Quantitative RT-PCR gel showing knockdown of *TmCactin* by both dsRNAs (ds*TmCactin* and ds*TmCactin* #2) compared to the

control *EGFP* dsRNA. *TmL27α* was used as a loading control. (C and D) Similar to the first dsRNA, injection with ds*TmCactin*#2 also decreases larval survival rates after *E. coli* and *S. aureus* challenge compared to *EGFP* dsRNA control-injected larvae.

Figure S4. Quantitative RT-PCR confirms that *E. coli*- and *S. aureus*-induced expression of *Tene-1*, *Tene-4*, *Def-1*, *Def-2*, *Cole-1*, *Cole-2*, and *Att-1b* is reduced in larvae injected with ds*TmCactin*#2.

Supplementary Table 1. Top 100 blastp hits (all with E-values of 0.0) from TmCactin alignment to the NCBI nr database (last accessed Dec. 12, 2016).

Figure S1

A

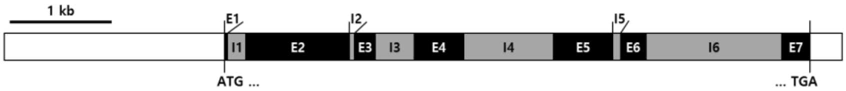

B

```
tcaacacagataaatatgaaatgatgtttttatcaagaaccttacaacgcagctatctactatacatatgttacgttacgttagttgatt
aatgaaataacaattcccaatttttttgaaatcccaacttttcaaacatacaaatcgacgctttgttctgggcgtaaatggtcgcaaaagg
acgctgtccctcttccactatctctttagctaaagccttagtaaccaacaacccatatccCttcttttttttcgttttctcgagtctgaac
catcccgatgtgtcccaagcagtgctttacggcccacgccaccatctcgtctccatttttcaaaaacactccataaccgccattactctc
gatcaacgaagctatataatcctgggatcccggatatttgtagggccacaccgaatcaatctgcgcagctgattccacatccaactccct
caagtaaactcccgtgggatgttcattcgaaaattcgctctttcccgcagctaacgcgtacatcaaatacggagcttcagttttgtcgac
gttcaactctttctcctcgaagatcgacatgacggtggaaacgtgtttctcgtgaacgcagaaaaacgtcgcctgcgggtttttgtagtc
gatgaaatttgtccgactcaagccttctcgcagatttgtacattttcgtccagagtgaatacgaatatgttgtagcagtaattctaaaa
agtgagttgtgcgttacttcctactgagcatgtcttacttacggtgaatagaaccacgatggtgccatctctctcccaactatcgttggg
tgacgtgaaactgatacaatcacgatatttcgccgatcgtttccagcgaatccccatgtgaatcaggcttaaaacgtgggggcccatcc
gacatgtttcttgtataattcggccaacgcaggcaaggtttcatccgcgatttcgcgtaaaatatcgttcgcgagtggagccatttattt
tgtgtttaccccaccacgcgacgataacccctctaccttcaaaacagtagaactgaggacaagtttcttggattttgacgtgacaagaac
ggaaacaaaagcaaattattgccgcttaaattacaataactttattgataaaaataacactaagagatgacatgttaaaacgttagcgta
ggaagcagcctggtaattaaaaacaaaaacaatatgcttgttagtactgaaggataaactgggaaccatcccgaagaaagttcaataaga
Ttacagccttgtttaatcgaaaggaccactttgtggccccaagtaaacattcaggacgtgtcgcggacaccagggcccgtccctaatgtt
aaaaaactaacataatgaacaaatttacaaatatcatttagtttgagcacttatttcccattgtgagttggaggcgttgtgattggtcgt
ttaaatagcaggatgtgtggttctgcaaagttcaaaatcatatttcttgcaataagggccgatcgtccggcactcacctggagaatgcgt
catataatgcacccaacctttactctgttgtactccgatacctcgccattcctcttccgacatcagatgagttttcggcaccaactttac
Catttctttgggtaaaacgacgtgtctgacgaaaacaaaacgaattaatttgcgtctcacccttggtgccggaaaatcccggaaaaatct
gctcaccggtattcgtactcgtcgtcgtagtacttatcggagtagtaaatgtttctctgcatttcctcgattttcatgcgattcatggaa
cctgctgtgctttttaaaccgatagaaataaatgaaaattcgatttttttttcgtttttcaccacaaaaactttcacttctttcaaaccg
acaaaagttttgattgtgttgccaaccgtgcaaaccactaaacaccaacttgattcgcaatcccaaggtcgcacccatctggcttacgat
tagtttataatagttgaattacgcttcgacaaacctttcacggtcctcaaatcgatcacggataaagaatcgttccttaatatatttacg
agaaatagaaagttacgagacgtaataccaacttgacaaagttcaacgttatgttgacgtttcactaacctataaaccaaaacaagagtt
ttgtccagataaaATGCCAAGAAGTGACCATTCTCGACGACACAGGTGAGTTTTAGTTATAATTTTATTTGAGCGTTGCTAAGAAATGTA E1
TGTAATGATAGGTCTTCTGGCCGTCCTCACGATCGTTCGTCGGACACGGGCGATAGCGAAGATGAGAGACAAAAATCGAGGAAACGAGGA
AACTTGGATAAATTTAAGAAAAGAGATCGTAGCTCTAGCGAGAGATCGTCGAAAAAGAAAGAGAAAAAGCACAGATTTCGTGACTCAGAT
TCAGATTCGAGCAGCAGCTCGTCTGATGATTCATTAATCCTCCTTCAGAAGCTCAAAGAAGAAAGACTGAAGGCTCTTGATGAAAGAAAG
CGTGAAAAAGAATTGAAAAAAGCGACGGAAACGCCAGAAGAGAAGAGATTGAGGCGCTTGTCAAAAAAACAGGCTAAAGAGAGAAAGCGC
AAAGAGCGCATGGGGTGGGATAATGAATACCTTCATTACACCAACACTGATAATCCGTTCGGCGACGGCAATTTGCTGTCAACTTTTGTC
TGGACGAAAAAATTGGCCAAGGAAGGACTTGGCAACGTGAGCCAGGAAGAACTAGAAGCGCGAAATCGCCACAAACAGGAAGAAAACAAA
CGCGAATTGGAGAAAGTGAAGAAGAGAAGAATAGAACGAGAGCTGGAGAGGCAAAAGAGAGAAGAGGAGATGCAACTGTTGCAGCGTAGC E2
AAAGAAGCGGCGCAATTTGAAGAGTGGGAACGTCAAGAGGATCAGTTCCACTTGGAACAAGCGAGGCTGAGATCGCACATTCGCATTCAG
GACGGCCGAGCCAAACCCATCGACTTGTTGGCGAAATACATAAGCGCGGAAGAAGAGGTCGACGCGGTGGAAATGCACGAGCCGTACACG
TACCTGAACGGTCTGCACATCAAAGATTTGGAAGATCTAGTTGAGGACATAAAAGTCTACGAAGAACTGGAACGGGGGAAGAACCTAGAT
TACTGGAACGACATCACGGTCATCGTAGAGGACGAGCTACAAAAACTGCGAAAACTAGAAAAACAAAATGAATTCGATATGGGAGTGAAC
AGGCGGGAAGGCATCAACCAGGCTGTCGCCGCTGACGTGACGTCGGTGTTCAAAGGGAAGACGTCGGCACAACTGGCGGCGCTGCAGAAG
CAGATCGAGAGCAAAATCAACGGCAAAGCGGACGGAATCGACATAGGCTATTGGGAATCGTTGCTGTCACAGCTGAAGGGTTGGAAGTCT
TTTTTTATCAGTTTTGTTTTGTAAACCTTGAACTGTTACAGCTCACATGGCGAGGGCGCGGTTGAGGGACCGACATCAGGACAGTCTCAG
GAGGAAATTGCAAGTCCTCAAAGCTGAACAAGCCGTTACTGTGCCGGCAGCAGAAGAAAATACCACTCCTGTGGCGGAGACGCATGCGCA E3
AATGGAGCCAGAGCCCAAACCCGGCACCTCGAATGAAGAAGAACAAATCAGCGACAACGAGAAGTATGGGATGCGGATATTTTTATTTTT
CATTGCTCAGAAAATCCTAACCTCAGTTTTGAATTTGTTACCTCACTTAAAAAAAAAAAAATACGATTTTTATTTAATCGATACAGGATA
TTATAAGTTTCTCGATAGATATGATGATTTCAAATTTTACATTTAAATTTTAGCGAATGCAGAACAGATTGTTGTATTTTTTATTGTTTC
CGATGATGAGAGGTAAGTGAGGTTATGTCATATTTTTTTAAATTCATGTAAGTGGAAATCGTTATATCTTACACAAAAAAGCTGTTGCGC
AAGTCTATTGAAATGTTATTATTTCAATTGATTGACTCATAAATTATGATTATGAGATTTTGCACACTCGTAAAATAAAATGCTTTCAGA
ACCGCCGAGGAAAACGCGAACGACATCTTAAACGAGTACTTCGACGCTTACGAAGCGGGCGGCTACAGCCCCAAGTTCTTACCCCCCGAG
GCCATCGAACCCGGCACCATAGTCGTAACCGAAGAAGACGACTCAAAACGTCTTGAATTTGCGCGAATGCAAGTCTGCGGCAGCGGCAAG
AAGGTCGAAAACGTGATAACGAAAGAAGAACTCCTTCTGCAACGCGAAGCCCGAAAAGGAATGGGCGACGACGAGGCGCAGTTTTCCGTA E4
GAAGCGGCCCTCGACAACCAGGTCTACCTCTGGTCGGACAAATACCGACCGAGAAAACCGAGATATTTCAACAGAGTCCACACAGGTTTC
GAGTGGAACAAATACAATCAAACCCATTACGACATGGACAATCCTCCGCCGAAGATCGTGCAAGGGTACAAATTTAACATTTTCTACCCA
GACCTGATCGACAAAAACAGCACACCCGAGTATTTCTTGGTAAGTTGGTGGAAGGGTTGAAGGGTCAGATGTTTGACGGTTTTGTTTACA
GACGCCCTGTCCGGAAAATCGGGAGTTTGCGATTTTGAGGTTCCACGCTGGGCCACCCTACGAAGACATTGCGTTCAAAATTGTCAACAG
GGAATGGGAGTACTCGTACAAGAGGGGGTTCAGGTGTCAGTTCCACAACAACATTTTTCAGTTGTGGTTCCACTTCAAGAGGTACCGGTA
CAGAAGATAATTCAGCTTTCCAATTTTACATCGTGTAAATACAGTTATATTTAAGATTATATAAAGAATCCAATTTTACGAGGTTTTTAT
TTATTTGACTTAACCCAGCGTTGTGCAAGACAAGAACGAATTATTTACAAGATCAGTTACTTTTTGAAAAAAAAATCAATTGGCTCAAGA
ACGTCACTGGGGCAACGTTCTTTCGAAGGAAAGTTCTTTCCGTGGTCAAATCCTTAAAAGTGTATCCGTAGGTGGTGAATTACCCTTGAG
CCTCCTAATATAGGTTTGTTGTTAGTCATGTTCGCAGTCGTCTGGTCGATTTTGCGCGTAGGTAGGCATAAAGAAGTAACCGCTACCTAA
CAAGTTGCATACCAGTATTACCGGAGCGACGTCGTTGTTTGTATCGAGCGCGCTTATTATTTTTTCTAGTCCTCGCGCGCCCGTTTATTT
ACTTTATTTATTTACAAGTGTACGATACAGTGAACCAATGTGAAAGTGCAACTTATGACGCTAAAGACTGAGAACAAGACGACCAAACGA
AAATCGCGCCCTTTCCTGGTACTGCCTCTTCTAGAGAACCTCGCGCTGAAGAAACTGTCCAAAATGCCCAAAAACACAAGCTTGAAGTTG
TTCACGAAGACGTGGCCCCCGAAGAGGGACGGCAAGAAGGAAGAGAGACCCTTGACGGCGACGTTAGACAAGCCCACCGTCCTCAACATC
AGCCTCATCGGAATAATCGACATGAGCACCATGAGAAAAGAGTTCTTGAACATATCGAACCTGTGCCGGTTCCCTCTCTCGTTGGCCATC
GCCAAGAGAAAGAAGACGGCGGTGATCAACCGCCCCCTCAGCCTTCCATCTCCGGAGAAGCACAAAACTCGCAAGAGCAAGCGACCCGAA
ACGAGCGAGGACAGCTACGAAGAGGAAGAGAGCAGCGTCAGCGTGAAAAGCGCCAAGACGAAGAACGGGACAGCCAAAGCGAAGAAGGTC E5
GACCTTCTAGCGAAGAAGAAGAGTTTGAGCGACGCCAACAACAACAACAAGACGTTCAAAATTCCACGCAAGCGTGTTGGCAATGTTGTT
AAGTTGGTGGATGTGAGCAACAAGAGCGATCTGGGCAAATCGACCAAACTGCCGGAGCCTTGCAGGACTTGTGGCAGACCTGACCAACCC
GAACGTTTCCACAGCCATCCTGTGACGCCACTCAAAGTCACCAAAAAGGCCGAGGAACCCCCCAAAAGTGAGTCCAATTATTGCAACCGG
CGCATCAGCGCTAAATTTAGCACGATTACTCACCTCGTCGCGTCGATTGCTTTGCAGTGAAGAGCACCGTCCAGAAACCGGTGGCGATGA
AGTACAAGTCGAAGCAACAGAACTCCGACAAGAATCTGCCACCACCGCCTCAGCGCAAACCCTCCCTGCCGGCTCAGAAAATCGTGCACA
GCCCCCCTAACGTGGGCACCGCCGCCACCCCCAGGGTGAAGTCGGCGAAGCGGACGCTCACTTGCTACATCTGCGGCAGGGAGTTCGGCA E6
CCGCCTCGCTCCACCTGCACGAGCCCAAGTGCCTCCAGGTACCCCCATTTCAACATACCACTTAAATTATTCCCAAACGGAACACCTTCG
TGCGGCGCGAGAAATATTTTCGTGCCGATAATCGCACCCCAATTTTGTGCTCGCATGTTCCCGCAGGAATCGAACGGTCTGACAGTTCTT
ACGTCGCATAAATATTAAATGATGTCCAAGTTCAAGACTGGATGCCATTGATAAAATGCTAATTGATGTAAATGTATGAGGAATCGATCA
TGGAAAATAATAAGCGGCGGGGGTTGAATCCCAAAGGCGACAACCTTGAACTTTCGATGGGGGTTTTTTGGTGGAAAAATACATTGAAGA
CGGTTTAAATTACAGTGTGAAGAGCTCAAAATATCTCGTTCTGGTTAATTCATGACAATCGATGACGATGATGGAATTATTAGAGGTGCG
TCACGCAATTGAACTGAAATTTCGCTCGGTAAATACGTTACAATTGAGGTAATTCGATAATCGTCCAATTCTACAACTGTTAGAATAAAA
TTGTATCACAAATCGATTGGAATTTTTTTAATAGCGTGACATTTGTGTCTGTCAAAATCAGTTGTTAGGTTGGTTAAATCANNNNNNNNN
NNNNNGTTTTCATAGGAGGAATTGATTGGGTACACAATGAACATGACACACAAAAGAACTAATTTGTTTTTCATTTTCACTAAATTACCC
CAAAACTATCCGAATTCTTTGAAAGTAATTAGGGCAACAGTGACTGATGACTGTAGACATGTTTGTAGTGTTTTCGTAAAAAAATTCTTC
AGCGGATTGACCAGAGGCGGTCTTCGAGACTATCAAATCGGTGAAATTTTAACATGCACGAGTGTTTTACAATTTAAATCGCCGTGGCAT
TTCACCCTTGAAAGCACCATATTTCAACTGGTACCCTAATTTTTTTCCCCTGGACTAGCAAGACAGTAAACCACCTTGAAACGTACGAAA
GCAACAAAGTAGCACAAAACTGCTGCATCGTTAACAGTTGCATGTTGATAAGCCACTACTCTGTCTTATCTTGATTTCGTGAAAGAATTG
ATTCTTCGACAAACACCGGGAAAATCCCGCAAGAGTTTCGCAAGACACACAAATTAAGTCGCCTTAAATTAATCACACAACGACAGATCC
CAAACGTCCTTCACGCCCCGTAATCCATCAAACCATTCCGCTGGAAAGCGATCATAAAAACTGACCTACAATGCGGAATATATTAACCCA
TATGCGGTCGAGCCGCACCGCCAAGTCGGCTAATCAAAACCTCGTAACTCCCGACGCAACCCCCATAATTTGCTAACGACACGGCGACCT
TTAACGACCTACTTGGCGGTCTTTGCAGAGATGGAACGGGAGAATGCCGGCTTGCCGACGCACTTGCAGAGGAAGCCCCCGGTCAAGCC
GGACCACCCTCTCACCCCAGAAGAATGGAACAAGTTCGCCTGGGAGTCCTCTCAGGTGAGTGTCAAGAAAATGTGCACACGACCTGGACA E7
AATCGATCGGTTTTTGAGCGACAATTATCGTGAAAGCTGTACAAGCACTGGGCAACACGCCAATGACGAGTTATGGGTCGACGAGGGTCC
AAAACACCCAAATTTAAATCGTTCGAATGATTGAtggtttttcgttgttatgcaaccaacAatacatttcgagcctcgcggccataaatc
ttaacaaactcgtgagttctatcagagcctttgaatattgtatgaaatcaattcgattttcgattagaacgattgcccttaacattgctt
cctgaacttccggcacattgatttccggtgtgcgtcagttgatttctttatttaaacaaaacatcgcgtgtcctgtcacacatcacaaaa
tggacggcccacgacgcattaattgttaaatattttcaatgttttcttttgatccggtttaaaagtgaatgtaaagtccattc
```

Figure S2

```
ATG CCA AGA AGT GAC CAT TCT CGA CGA CAC AGG TCG AAA AAG AAA GAG AAA AAG CAC AGA   60
 M   P   R   S   D   H   S   R   R   H   R   S   K   K   K   E   K   K   H   R    20

TTT CGT GAC TCA GAT TCA GAT TCG AGC AGC AGC TCG TCT GAT GAT TCA TTA ATC CTC CTT  120
 F   R   D   S   D   S   D   S   S   S   S   S   S   D   D   S   L   I   L   L    40

CAG AAG CTC AAA GAA GAA AGA CTG AAG GCT CTT GAT GAA AGA AAG CGT GAA AAA GAA TTG  180
 Q   K   L   K   E   E   R   L   K   A   L   D   E   R   K   R   E   K   E   L    60

AAA AAA GCG ACG GAA ACG CCA GAA GAG AAG AGA TTG AGG CGC TTG TCA AAA AAA CAG GCT  240
 K   K   A   T   E   T   P   E   E   K   R   L   R   R   L   S   K   K   Q   A    80

AAA GAG AGA AAG CGC AAA GAG CGC ATG GGG TGG GAT AAT GAA TAC CTT CAT TAC ACC AAC  300
 K   E   R   K   R   K   E   R   M   G   W   D   N   E   Y   L   H   Y   T   N   100

ACT GAT AAT CCG TTC GGC GAC GGC AAT TTG CTG TCA ACT TTT GTC TGG ACG AAA AAA TTG  360
 T   D   N   P   F   G   D   G   N   L   L   S   T   F   V   W   T   K   K   L   120

                  dsTmCactin#2
GCC AAG GAA GGA CTT GGC AAC GTG AGC CAG GAA GAA CTA GAA GCG CGA AAT CGC CAC AAA  420
 A   K   E   G   L   G   N   V   S   Q   E   E   L   E   A   R   N   R   H   K   140

CAG GAA GAA AAC AAA CGC GAA TTG GAG AAA GTG AAG AAG AGA AGA ATA GAA CGA GAG CTG  480
 Q   E   E   N   K   R   E   L   E   K   V   K   K   R   R   I   E   R   E   L   160

GAG AGG CAA AAG AGA GAA GAG GAG ATG CAA CTG TTG CAG CGT AGC AAA GAA GCG GCG CAA  540
 E   R   Q   K   R   E   E   E   M   Q   L   L   Q   R   S   K   E   A   A   Q   180

TTT GAA GAG TGG GAA CGT CAA GAG GAT CAG TTC CAC TTG GAA CAA GCG AGG CTG AGA TCG  600
 F   E   E   W   E   R   Q   E   D   Q   F   H   L   E   Q   A   R   L   R   S   200

CAC ATT CGC ATT CAG GAC GGC CGA GCC AAA CCC ATC GAC TTG TTG GCG AAA TAC ATA AGC  660
 H   I   R   I   Q   D   G   R   A   K   P   I   D   L   L   A   K   Y   I   S   220

   qPCR primers
GCG GAA GAA GAG GTC GAC GCG GTG GAA ATG CAC GAG CCG TAC ACG TAC CTG AAC GGT CTG  720
 A   E   E   E   V   D   A   V   E   M   H   E   P   Y   T   Y   L   N   G   L   240

CAC ATC AAA GAT TTG GAA GAT CTA GTT GAG GAC ATA AAA GTC TAC GAA GAA CTG GAA CGG  780
 H   I   K   D   L   E   D   L   V   E   D   I   K   V   Y   E   E   L   E   R   260

GGG AAG AAC CTA GAT TAC TGG AAC GAC ATC ACG GTC ATC GTA GAG GAC GAG CTA CAA AAA  840
 G   K   N   L   D   Y   W   N   D   I   T   V   I   V   E   D   E   L   Q   K   280

CTG CGA AAA CTA GAA AAA CAA AAT GAA TTC GAT ATG GGA GTG AAC AGG CGG GAA GGC ATC  900
 L   R   K   L   E   K   Q   N   E   F   D   M   G   V   N   R   R   E   G   I   300

AAC CAG GCT GTC GCC GCT GAC GTG ACG TCG GTG TTC AAA GGG AAG ACG TCG GCA CAA CTG  960
 N   Q   A   V   A   A   D   V   T   S   V   F   K   G   K   T   S   A   Q   L   320

GCG GCG CTG CAG AAG CAG ATC GAG AGC AAA ATC AAC GGC AAA GCG GAC GGA ATC GAC ATA 1020
 A   A   L   Q   K   Q   I   E   S   K   I   N   G   K   A   D   G   I   D   I   340

GGC TAT TGG GAA TCG TTG CTG TCA CAG CTG AAG GCT CAC ATG GCG AGG GCG CGG TTG AGG 1080
 G   Y   W   E   S   L   L   S   Q   L   K   A   H   M   A   R   A   R   L   R   360

GAC CGA CAT CAG GAC AGT CTC AGG AGG AAA TTG CAA GTC CTC AAA GCT GAA CAA GCC GTT 1140
 D   R   H   Q   D   S   L   R   R   K   L   Q   V   L   K   A   E   Q   A   V   380

                                                 dsTmCactin
ACT GTG CCG GCA GCA GAA GAA AAT ACC ACT CCT GTG GCG GAG ACG CAT GCG CAA ATG GAG 1200
 T   V   P   A   A   E   E   N   T   T   P   V   A   E   T   H   A   Q   M   E   400

CCA GAG CCC AAA CCC GGC ACC TCG AAT GAA GAA GAA CAA ATC AGC GAC AAC GAG AGA ACC 1260
 P   E   P   K   P   G   T   S   N   E   E   E   Q   I   S   D   N   E   R   T   420

GCC GAG GAA AAC GCG AAC GAC ATC TTA AAC GAG TAC TTC GAC GCT TAC GAA GCG GGC GGC 1320
 A   E   E   N   A   N   D   I   L   N   E   Y   F   D   A   Y   E   A   G   G   440

TAC AGC CCC AAG TTC TTA CCC CCC GAG GCC ATC GAA CCC GGC ACC ATA GTC GTA ACC GAA 1380
 Y   S   P   K   F   L   P   P   E   A   I   E   P   G   T   I   V   V   T   E   460

GAA GAC GAC TCA AAA CGT CTT GAA TTT GCG CGA ATG CAA GTC TGC GGC AGC GGC AAG AAG 1440
 E   D   D   S   K   R   L   E   F   A   R   M   Q   V   C   G   S   G   K   K   480

GTC GAA AAC GTG ATA ACG AAA GAA GAA CTC CTT CTG CAA CGC GAA GCC CGA AAA GGA ATG 1500
 V   E   N   V   I   T   K   E   E   L   L   L   Q   R   E   A   R   K   G   M   500

GGC GAC GAC GAG GCG CAG TTT TCC GTA GAA GCG GCC CTC GAC AAC CAG GTC TAC CTC TGG 1560
 G   D   D   E   A   Q   F   S   V   E   A   A   L   D   N   Q   V   Y   L   W   520

TCG GAC AAA TAC CGA CCG AGA AAA CCG AGA TAT TTC AAC AGA GTC CAC ACA GGT TTC GAG 1620
 S   D   K   Y   R   P   R   K   P   R   Y   F   N   R   V   H   T   G   F   E   540

TGG AAC AAA TAC AAT CAA ACC CAT TAC GAC ATG GAC AAT CCT CCG CCG AAG ATC GTG CAA 1680
 W   N   K   Y   N   Q   T   H   Y   D   M   D   N   P   P   P   K   I   V   Q   560

GGG TAC AAA TTT AAC ATT TTC TAC CCA GAC CTG ATC GAC AAA AAC AGC ACA CCC GAG TAT 1740
 G   Y   K   F   N   I   F   Y   P   D   L   I   D   K   N   S   T   P   E   Y   580

TTC TTG AAG AGG GAC GGC AAG AAG GAA GAG AGA CCC TTG ACG GCG ACG TTA GAC AAG CCC 1800
 F   L   K   R   D   G   K   K   E   E   R   P   L   T   A   T   L   D   K   P   600

ACC GTC CTC AAC ATC AGC CTC ATC GGA ATA ATC GAC ATG AGC ACC ATG AGA AAA GAG TTC 1860
 T   V   L   N   I   S   L   I   G   I   I   D   M   S   T   M   R   K   E   F   620

TTG AAC ATA TCG AAC CTG TGC CGG TTC CCT CTC TCG TTG GCC ATC GCC AAG AGA AAG AAG 1920
 L   N   I   S   N   L   C   R   F   P   L   S   L   A   I   A   K   R   K   K   640

ACG GCG GTG ATC AAC CGC CCC CTC AGC CTT CCA TCT CCG GAG AAG CAC AAA ACT CGC AAG 1980
 T   A   V   I   N   R   P   L   S   L   P   S   P   E   K   H   K   T   R   K   660

AGC AAG CGA CCC GAA ACG AGC GAG GAC AGC TAC GAA GAG GAA GAG AGC AGC GTC AGC GTG 2040
 S   K   R   P   E   T   S   E   D   S   Y   E   E   E   E   S   S   V   S   V   680

AAA AGC GCC AAG ACG AAG AAC GGG ACA GCC AAA GCG AAG AAG GTC GAC CTT CTA GCG AAG 2100
 K   S   A   K   T   K   N   G   T   A   K   A   K   K   V   D   L   L   A   K   700

AAG AAG AGT TTG AGC GAC GCC AAC AAC AAC AAC AAG ACG TTC AAA ATT CCA CGC AAG CGT 2160
 K   K   S   L   S   D   A   N   N   N   N   K   T   F   K   I   P   R   K   R   720

GTT GGC AAT GTT GTT AAG TTG GTG GAT GTG AGC AAC AAG AGC GAT CTG GGC AAA TCG ACC 2220
 V   G   N   V   V   K   L   V   D   V   S   N   K   S   D   L   G   K   S   T   740

AAA CTG CCG GAG CCT TGC AGG ACT TGT GGC AGA CCT GAC CAA CCC GAA CGT TTC CAC AGC 2280
 K   L   P   E   P   C   R   T   C   G   R   P   D   Q   P   E   R   F   H   S   760

CAT CCT GTG ACG CCA CTC AAA GTC ACC AAA AAG GCC GAG GAA CCC CCC AAA GTG AAG AGC 2340
 H   P   V   T   P   L   K   V   T   K   K   A   E   E   P   P   K   V   K   S   780

ACC GTC CAG AAA CCG GTG GCG ATG AAG TAC AAG TCG AAG CAA CAG AAC TCC GAC AAG AAT 2400
 T   V   Q   K   P   V   A   M   K   Y   K   S   K   Q   Q   N   S   D   K   N   800

CTG CCA CCA CCG CCT CAG CGC AAA CCC TCC CTG CCG GCT CAG AAA ATC GTG CAC AGC CCC 2460
 L   P   P   P   P   Q   R   K   P   S   L   P   A   Q   K   I   V   H   S   P   820

CCT AAC GTG GGC ACC GCC GCC ACC CCC AGG GTG AAG TCG GCG AAG CGG ACG CTC ACT TGC 2520
 P   N   V   G   T   A   A   T   P   R   V   K   S   A   K   R   T   L   T   C   840

TAC ATC TGC GGC AGG GAG TTC GGC ACC GCC TCG CTC CAC CTG CAC GAG CCC AAG TGC CTC 2580
 Y   I   C   G   R   E   F   G   T   A   S   L   H   L   H   E   P   K   C   L   860

CAG AGA TGG GAA CGG GAG AAT GCC GGC TTG CCG ACG CAC TTG CAG AGG AAG CCC CCG GTC 2640
 Q   R   W   E   R   E   N   A   G   L   P   T   H   L   Q   R   K   P   P   V   880

AAG CCG GAC CAC CCT CTC ACC CCA GAA GAA TGG AAC AAG TTC GCC TGG GAG TCC TCT CAG 2700
 K   P   D   H   P   L   T   P   E   E   W   N   K   F   A   W   E   S   S   Q   900

GTG AGT GTC AAG AAA ATG TGC ACA CGA CCT GGA CAA ATC GAT CGG TTT TTG AGC GAC AAT 2760
 V   S   V   K   K   M   C   T   R   P   G   Q   I   D   R   F   L   S   D   N   920

TAT CGT GAA AGC TGT ACA AGC ACT GGG CAA CAC GCC AAT GAC GAG TTA TGG GTC GAC GAG 2820
 Y   R   E   S   C   T   S   T   G   Q   H   A   N   D   E   L   W   V   D   E   940

GGT CCA AAA CAC CCA AAT TTA AAT CGT TCG AAT GAT TGA                             2833
 G   P   K   H   P   N   L   N   R   S   N   D   *                              952
```

Figure S3

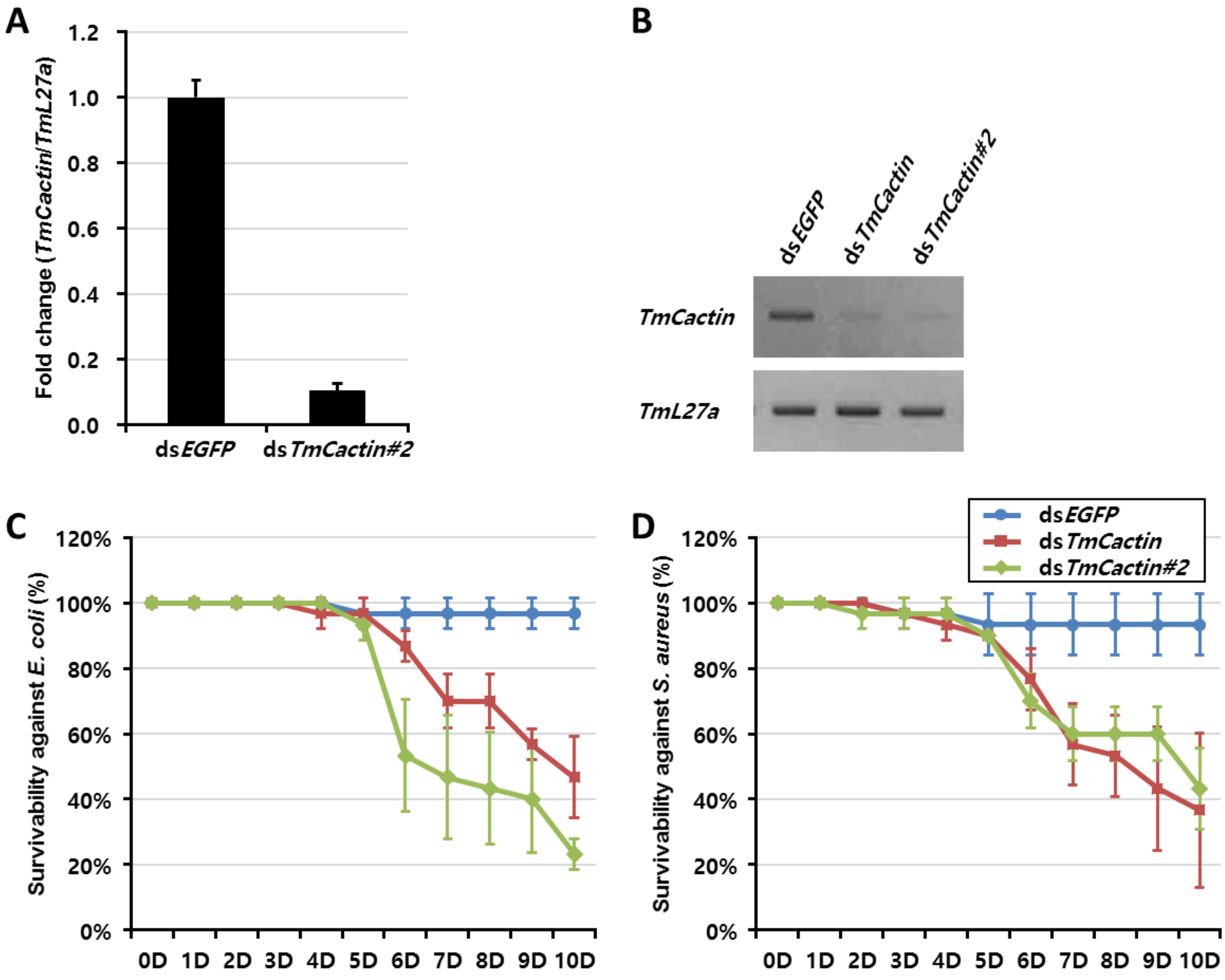
A
Fold change (TmCactin/TmL27a)
1.2
1.0
0.8
0.6
0.4
0.2
0.0
dsEGFP
dsTmCactin#2
B
dsEGFP
dsTmCactin
dsTmCactin#2
TmCactin
TmL27a
C
Survivability against E. coli (%)
120%
100%
80%
60%
40%
20%
0%
0D 1D 2D 3D 4D 5D 6D 7D 8D 9D 10D
D
Survivability against S. aureus (%)
120%
100%
80%
60%
40%
20%
0%
0D 1D 2D 3D 4D 5D 6D 7D 8D 9D 10D
dsEGFP
dsTmCactin
dsTmCactin#2

Figure S4

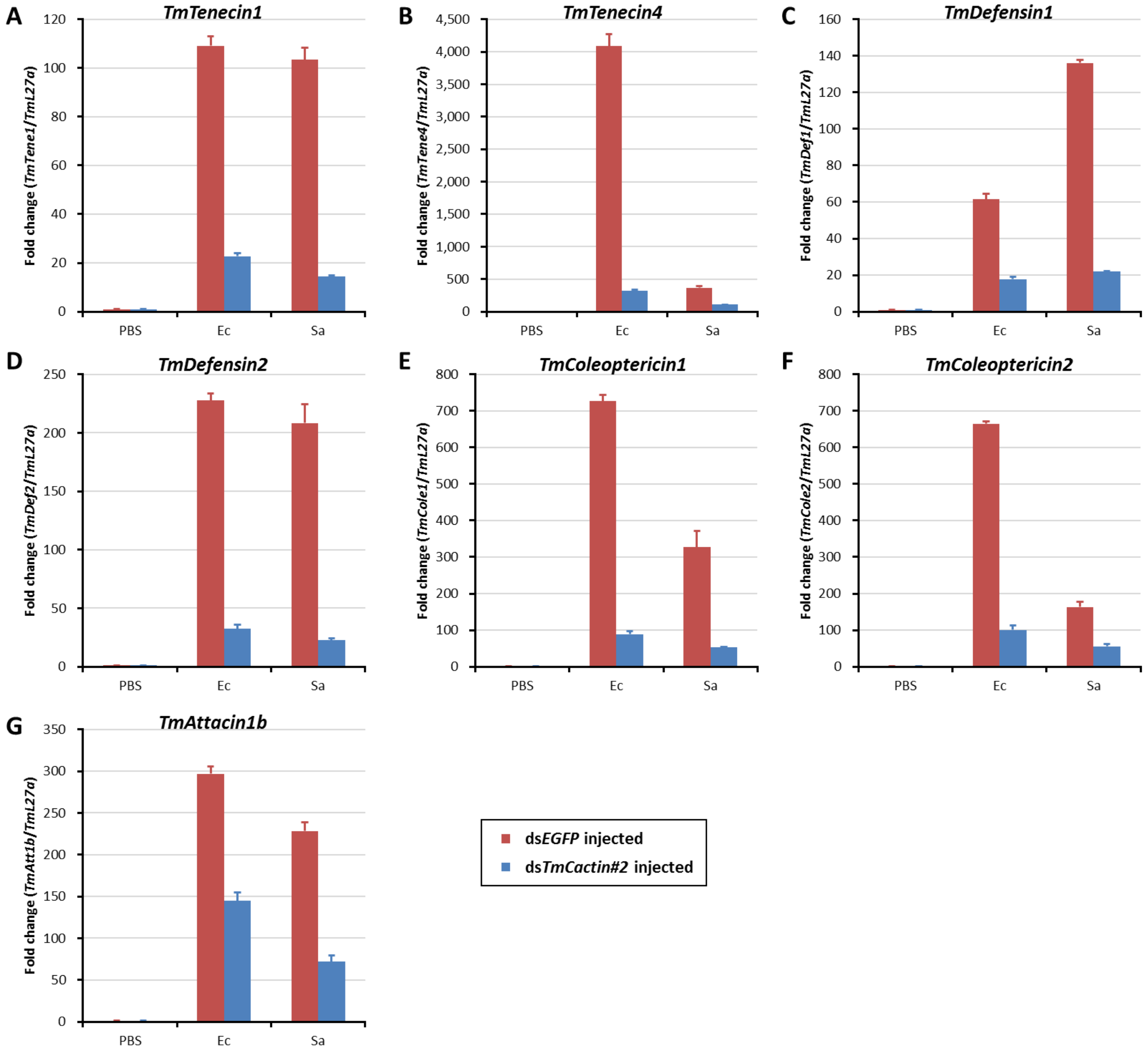
A
TmTenecin1
Fold change (TmTene1/TmL27a)
0
20
40
60
80
100
120
PBS
Ec
Sa
B
TmTenecin4
Fold change (TmTene4/TmL27a)
0
500
1,000
1,500
2,000
2,500
3,000
3,500
4,000
4,500
PBS
Ec
Sa
C
TmDefensin1
Fold change (TmDef1/TmL27a)
0
20
40
60
80
100
120
140
160
PBS
Ec
Sa
D
TmDefensin2
Fold change (TmDef2/TmL27a)
0
50
100
150
200
250
PBS
Ec
Sa
E
TmColeoptericin1
Fold change (TmCole1/TmL27a)
0
100
200
300
400
500
600
700
800
PBS
Ec
Sa
F
TmColeoptericin2
Fold change (TmCole2/TmL27a)
0
100
200
300
400
500
600
700
800
PBS
Ec
Sa
G
TmAttacin1b
Fold change (TmAtt1b/TmL27a)
0
50
100
150
200
250
300
350
PBS
Ec
Sa
dsEGFP injected
dsTmCactin#2 injected

Supplementary Table 1. Top 100 blastp hits (all with E-values of 0.0) from TmCactin alignment to the NCBI nr database (last accessed Dec. 12, 2016).

**Species**	**Common Name/Organism**	**GenBank Accession #**	**Sequence Description**	**Query Coverage**	**Alignment Length**	**Percent Similarity**	**Percent Identity**
Tribolium castaneum	Red flour beetle	XP_008196004.1	Predicted cactin	60%	600	91%	87%
Anoplophora glabripennis	Long-horned beetle	XP_018564952.1	Predicted cactin	57%	545	92%	86%
Agrilus planipennis	Emerald ash borer	XP_018333554.1	Predicted cactin	56%	540	88%	79%
Cyphomyrmex costatus	Fungus-growing ant	KYM93587.1	Uncharacterized protein C19orf29	97%	1070	63%	50%
Dendroctonus ponderosae	Bark beetle	ENN71807.1	hypothetical protein YQE_11541	59%	574	88%	80%
Pogonomyrmex barbatus	Red ant	XP_011629906.1	Predicted cactin	57%	547	86%	73%
Nicrophorus vespilloides	Burying beetle	XP_017776307.1	Predicted cactin	57%	549	88%	77%
Atta colombica	Leafcutter ant	KYM76580.1	Uncharacterized protein C19orf29	90%	985	62%	49%
Vollenhovia emeryi	Myrmicine Japanese ant	XP_011861876.1	Predicted cactin	57%	547	85%	74%
Atta cephalotes	Leafcutter ant	XP_012057115.1	Predicted cactin	57%	547	85%	73%
Atta colombica	Leafcutter ant	XP_018056418.1	Predicted cactin isoform X1	57%	547	85%	73%
Acromyrmex echinatior	Panamanian leafcutter ant	XP_011064726.1	Predicted cactin	57%	547	85%	73%
Trachymyrmex cornetzi	Fungus-growing ant	XP_018357566.1	Predicted cactin isoform X1	57%	547	85%	73%
Pediculus humanus corporis	Body louse	XP_002428222.1	Paramyosin, putative	57%	549	85%	73%
Trachymyrmex cornetzi	Fungus-growing ant	XP_018357567.1	Predicted cactin isoform X2	57%	547	85%	73%
Linepithema humile	Argentine ant	XP_012233515.1	Predicted cactin	57%	546	86%	73%
Dinoponera quadriceps	ant	XP_014477760.1	Predicted cactin	57%	550	86%	74%
Harpegnathos saltator	Indian jumping ant	XP_011152612.1	Predicted cactin	57%	551	86%	72%
Harpegnathos saltator	Indian jumping ant	EFN75758.1	Uncharacterized protein C19orf29	57%	551	86%	72%
Cerapachys biroi	Clonal raider ant	XP_011349145.1	Predicted cactin	57%	549	85%	73%
Camponotus floridanus	Florida carpenter ant	EFN62654.1	Uncharacterized protein C19orf29	80%	545	86%	72%
Eufriesea mexicana	Orchid bee	XP_017752636.1	Predicted cactin	57%	546	87%	75%

Habropoda laboriosa	Blueberry bee	XP_017790710.1	Predicted cactin	57%	549	87%	75%
Camponotus floridanus	Florida carpenter ant	XP_011264558.1	Predicted cactin	57%	547	84%	72%
Ceratina calcarata	Carpenter bee	XP_017883562.1	Predicted cactin isoform X2	57%	545	86%	75%
Bombus impatiens	Bumble bee	XP_003491995.1	Predicted Cactin	57%	546	86%	75%
Dufourea novaeangliae	Pickerel bee	XP_015439954.1	Predicted cactin	57%	550	86%	74%
Apis cerana	Indian honey bee	XP_016910227.1	Predicted cactin	57%	546	86%	75%
Dufourea novaeangliae	Pickerel bee	KZC06580.1	Uncharacterized protein C19orf29	57%	550	86%	74%
Apis florea	Red dwarf honey bee	XP_012345914.1	Predicted cactin isoform X2	57%	546	86%	75%
Bombus terrestris	Buff-tailed bumble bee	XP_003395486.1	Predicted cactin	57%	546	86%	75%
Lasius niger	Black garden ant	KMQ94182.1	Predicted cactin isoform 2	57%	545	86%	74%
Apis florea	Red dwarf honey bee	XP_003696278.1	Predicted cactin isoform X1	57%	546	86%	75%
Trachymyrmex zeteki	Fungus-growing ant	XP_018310944.1	Predicted cactin isoform X1	57%	545	86%	73%
Apis florea	Red dwarf honey bee	XP_012345915.1	Predicted cactin isoform X3	57%	546	86%	75%
Trachymyrmex zeteki		KYQ50290.1	Uncharacterized protein C19orf29	57%	545	86%	73%
Megachile rotundata	Alfalfa leafcutter bee	XP_003700357.1	Predicted cactin	57%	549	86%	74%
Wasmannia auropunctata	Little fire ant	XP_011696940.1	Predicted cactin	57%	547	85%	73%
Polistes dominula	European paper wasp	XP_015180263.1	Predicted cactin	57%	547	86%	74%
Trachymyrmex septentrionalis	Fungus-growing ant	XP_018342983.1	Predicted cactin	57%	547	85%	73%
Orussus abietinus	Wood wasp	XP_012275920.1	Predicted cactin	57%	545	86%	74%
Atta colombica	Leafcutter ant	XP_018056419.1	Predicted cactin isoform X2	54%	524	85%	74%
Apis mellifera	European honey bee	XP_016767776.1	Predicted low quality protein cactin	57%	546	86%	74%
Monomorium pharaonis	Pharaoh ant	XP_012529842.1	Predicted cactin	57%	548	85%	73%
Athalia rosae	Turnip sawfly	XP_012258533.1	Predicted cactin	57%	550	85%	74%
Acromyrmex echinatior	Leaf-cutter ant	EGI59105.1	Uncharacterized protein C19orf29	54%	524	85%	74%

Polistes canadensis	Red paper wasp	XP_014599721.1	Predicted cactin-like	57%	548	86%	74%
Ceratina calcarata	Small carpenter bee	XP_017883559.1	Predicted cactin isoform X1	57%	573	82%	71%
Solenopsis invicta	Fire ant	XP_011172320.1	Predicted cactin	57%	547	85%	72%
Neodiprion lecontei	Pine sawfly	XP_015509948.1	Predicted cactin	57%	550	85%	74%
Polistes canadensis	Red paper wasp	XP_014609071.1	Predicted cactin-like	57%	548	86%	74%
Trachymyrmex septentrionalis	Fungus-growing ant	KYN38820.1	Uncharacterized protein C19orf29	57%	566	84%	71%
Cephus cinctus	Wheat stem sawfly	XP_015599202.1	Predicted cactin	57%	550	85%	73%
Clastoptera arizonana	Arizona spittle but	JAS19691.1	Hypothetical protein g.5547	57%	544	84%	73%
Acyrthosiphon pisum	Pea aphid	XP_001952287.2	Predicted cactin	56%	557	80%	68%
Diuraphis noxia	Russian wheat aphid	XP_015379807.1	Predicted cactin	56%	557	81%	68%
Fopius arisanus	Tephritid fruit fly parasitoid	XP_011307236.1	Predicted cactin	57%	562	83%	72%
Bemisia tabaci	Tobacco whitefly	XP_018916407.1	Predicted cactin	57%	550	82%	71%
Cyphomyrmex costatus	Fungus-growing ant	XP_018405760.1	Predicted cactin	54%	524	85%	74%
Trachymyrmex zeteki	Fungus-growing ant	XP_018310945.1	Predicted cactin isoform X2	54%	522	86%	74%
Homalodisca liturata	Smoketree sharpshooter	JAT00701.1	Hypothetical protein g.11009	57%	544	85%	75%
Diachasma alloeum	Wasp	XP_015123757.1	Predicted cactin	57%	548	84%	72%
Microplitis demolitor	Wasp	XP_008556943.1	Predicted cactin	57%	574	80%	70%
Apis dorsata	Giant honey bee	XP_006614047.1	Predicted cactin-like	57%	546	84%	73%
Nasonia vitripennis	Jewel wasp	XP_003425945.3	Predicted cactin	54%	523	81%	70%
Ceratosolen solmsi marchali	Fig wasp	XP_011502302.1	Predicted cactin	54%	525	81%	70%
Copidosoma floridanum	Wasp	XP_014214609.1	Predicted cactin	57%	548	80%	67%
Cimex lectularius	Bed bug	XP_014248225.1	Predicted low quality protein cactin	57%	544	80%	69%
Trichogramma pretiosum	Wasp	XP_014223848.1	Predicted low quality protein cactin	57%	544	78%	67%
Halyomorpha halys	Brown marmorated stink bug	XP_014290251.1	Predicted cactin	57%	545	82%	68%
Cuerna arida	Sharpshooter	JAS56516.1	Hypothetical protein g.16355	52%	499	84%	73%
Daphnia magna	Water flea	JAN43572.1	Cactin	57%	569	73%	59%
Daphnia magna	Water flea	JAN86127.1	Cactin	57%	569	73%	59%

Daphnia magna	Water flea	JAN52132.1	Cactin	57%	569	73%	59%
Daphnia magna	Water flea	JAN44925.1	Cactin	57%	569	73%	59%
Daphnia magna	Water flea	JAN46435.1	Cactin	57%	569	73%	59%
Daphnia magna	Water flea	KZS15704.1	Cactin	57%	562	74%	59%
Rhipicephalus microplus	Cattle tick	AIT40194.1	Cactin	58%	578	75%	60%
Amblyomma aureolatum	Tick	JAT94195.1	Putative cactin	57%	568	74%	60%
Amblyomma sculptum	Tick	JAU02627.1	Putative cactin	57%	567	74%	61%
Daphnia magna	Water flea	JAN58168.1	Cactin	57%	571	72%	58%
Lucilia cuprina	Sheep blowfly	KNC34812.1	Cactin	57%	549	72%	59%
Lipotes vexillifer	White-flag dolphin	XP_007460408.1	Predicted cactin	56%	555	72%	56%
Tursiops truncatus	Bottlenose dolphin	XP_004317108.1	Predicted low quality protein cactin	56%	555	72%	56%
Ixodes scapularis	Black-legged tick	XP_002409176.1	Conserved hypothetical protein	57%	580	73%	59%
Orcinus orca	Killer whale	XP_004286374.1	Predicted cactin	56%	553	71%	56%
Lottia gigantea	Sea snail	XP_009048707.1	Hypothetical protein LOTGIDRAFT_157867	55%	566	70%	58%
Litopenaeus vannamei	Whiteleg shrimp	AIZ03630.1	cactin	57%	575	74%	57%
Drosophila simulans	Fruitfly	XP_016040165.1	Uncharacterized protein Dsimw501_GD28616	58%	581	70%	56%
Sorex araneus	Common shrew	XP_004614888.1	Predicted cactin	55%	548	71%	56%
Drosophila melanogaster	Fruitfly	AAF66981.1	cactin	57%	571	70%	56%
Drosophila melanogaster	Fruitfly	NP_523422.4	cactin	57%	571	70%	56%
Drosophila melanogaster	Fruitfly	AER42874.1	LP09118p1	57%	571	70%	56%
Alligator mississippiensis	Alligator	KYO34526.1	cactin	59%	574	71%	57%
Geospiza fortis	Medium ground finch	XP_014165073.1	Predicted low quality protein cactin	56%	546	73%	58%
Microtus ochrogaster	Prarie vole	XP_005359049.1	Predicted cactin	58%	570	71%	56%
Lepidothrix coronata	Blue-crowned manakin/bird	XP_017692466.1	Predicted cactin	56%	549	73%	58%
Camelus bactrianus	Camel	XP_010965004.1	Predicted cactin	58%	579	72%	56%
Alligator sinensis	Alligator	XP_006027263.1	Predicted cactin	56%	548	72%	58%
Charadrius vociferus	Killdeer/bird	XP_009878904.1	Predicted cactin	56	549	73%	57%
